# An algorithm for effectively separating overlapping spectra of chlorophyll a and b in *in vivo* broadleaf leaves and the development of a low-cost non-destructive detector for their contents

**DOI:** 10.3389/fpls.2026.1887710

**Published:** 2026-09-03

**Authors:** Wei Sun, Yixin Xiao, Dong Li, Yao Zhang, Kaihua Wu, Fumin Wang, Yihuang Chen, Hao Wang, Mengdian Wang, Enze Wang, Jian Sun, Jiabin Shu

**Affiliations:** 1 College of Artificial Intelligence, Hangzhou Dianzi University, Hangzhou, China; 2 Institute of Agricultural Remote Sensing and Information Application, Zhejiang University, Hangzhou, China; 3 Zhejiang Negrow Technology Co., Ltd., Hangzhou, China

**Keywords:** Beer-Lambert law, CabD, component-specific leaf chlorophyll detection, low-cost spectral detection, spectral overlap separation

## Abstract

Chlorophyll a (Chla) and chlorophyll b (Chlb) in plant leaves play distinct physiological and ecological roles, but most portable chlorophyll meters, such as SPAD meters, mainly provide relative chlorophyll indices rather than the individual contents of Chla and Chlb *in vivo* leaf conditions. This limitation restricts the acquisition of component-specific pigment information from living leaves and reduces the usefulness of field measurements for validating satellite-derived vegetation biochemical parameters. One key difficulty is that the spectral responses of the two pigments are highly overlapped *in vivo*, making it difficult to quantitatively characterize their individual absorption contributions and determine suitable sensitive bands for low-cost optical detection. In this study, a Beer–Lambert-inspired pseudo-absorption framework was developed to characterize and effectively separate the overlapping absorption-related spectral responses of Chla and Chlb in leaves of broadleaf species under *in vivo* conditions. Unlike conventional spectral-index-based approaches, the proposed method provided a physically interpretable empirical basis for sensitive-band selection. Using leaf hyperspectral data, 700 nm and 650 nm were identified as the sensitive bands for Chla and Chlb, respectively, while 900 nm was selected as the reference band for optical path normalization. The algorithm achieved RMSE values of 3.001 μg·cm^-2^ for Chla and 1.805 μg·cm^-2^ for Chlb. Based on the selected bands and optimized channel bandwidths, a low-cost, non-destructive detector for leaf Chla and Chlb contents, named CabD, was developed by integrating controllable spectral generation from multi-band light sources with photoelectric detection. Validation showed that CabD achieved RMSE values of 1.743 μg·cm^-2^ for Chla and 0.871 μg·cm^-2^ for Chlb, respectively, with relative accuracy values of 94.746% and 92.789%. *In situ* validation using 84 intact leaves yielded RMSE values of 2.245 and 1.530 μg·cm^-2^, with corresponding relative accuracies of 93.86% and 90.24% for Chla and Chlb, respectively. These results demonstrate that the Beer–Lambert-inspired pseudo-absorption framework can support a physically interpretable empirical strategy for sensitive-band selection in low-cost *in vivo* chlorophyll detector design. The developed CabD provides a practical tool for non-destructive acquisition of component-specific chlorophyll information and can support field monitoring and remote sensing validation of vegetation biochemical parameters.

## 1 Introduction

Chlorophyll a (Chla) and chlorophyll b (Chlb) are the primary pigments responsible for light absorption and energy conversion in green plants (Lichtenthaler, 1987). As key pigments involved in photosynthetic light harvesting, Chla and Chlb are closely associated with carbon assimilation and biomass accumulation in higher plants. Functionally, Chla serves as the dominant pigment for light capture and the conversion of light energy into chemical energy, while Chlb functions as an accessory pigment that enhances absorption under low-light conditions (Lichtenthaler, 1987; Taiz et al., 2015). Plants regulate both the absolute chlorophyll contents and the Chla/Chlb ratio to adapt to varying light environments. For instance, in several tropical crop species, shade-grown leaves exhibited higher total chlorophyll concentrations but lower Chla/Chlb ratios than leaves grown under full sunlight, although the magnitude of these responses differed among species (Johnston and Onwueme, 1998). Chlorophyll composition also varies across developmental stages; the Chla/Chlb ratio is approximately 2:1 during early leaf development and increases to about 3:1 upon leaf maturation (He et al., 2023). Environmental stresses further influence chlorophyll dynamics: heat stress induces chlorophyll degradation, altering total chlorophyll levels, while drought and water stress reduce chlorophyll content and decrease the Chla/Chlb ratio in both tolerant and sensitive species (Zhang and Tan, 2001; Guo et al., 2006; He et al., 2023). Given these fluctuations, accurate, component-specific quantification of Chla and Chlb contents is essential for evaluating plant physiological status. This need is particularly relevant for rapid and non-destructive chlorophyll monitoring across crop growth stages and phenological conditions, as emphasized in recent hyperspectral remote sensing studies (Guo et al., 2024).

However, existing non-destructive detectors for leaf chlorophyll status generally output relative chlorophyll indices, such as SPAD values from the Minolta SPAD-502 and chlorophyll content index (CCI) values from the Opti-Sciences CCM-200, rather than directly distinguishing individual components such as Chla and Chlb. Recent handheld chlorophyll detectors have attempted to estimate Chla, Chlb, and total chlorophyll content using low-cost sensing modules (Pan et al., 2025). Nevertheless, these detectors generally rely on only a few sensitive and reference wavelengths with predefined bandwidths to estimate chlorophyll content in transmission or reflection modes (Uddling et al., 2007; Mulero et al., 2022), and the effective separation of the overlapping spectral absorption features of Chla and Chlb *in vivo* remains insufficiently addressed in these devices. While this simplified spectral configuration enables cost-effective measurements, it also limits the capability to resolve overlapping absorption features of different chlorophyll components. A key physical challenge is that variations in leaf internal structure alter the effective optical path length of light–leaf interaction. Without an explicit mechanism to normalize this structural effect, effectively separating the highly overlapping *in vivo* absorption features of Chla and Chlb using only a few discrete bands becomes mathematically ill-posed. Moreover, compared with hyperspectral instruments, these detectors offer the advantage of reduced acquisition cost through the use of discrete-wavelength light sources (Mulero et al., 2022; Kamarianakis and Panagiotakis, 2023), which is consistent with recent evaluations showing that low-cost discrete-band optical sensing can provide a practical pathway for non-destructive chlorophyll assessment when wavelength selection and calibration are carefully considered (Lopin et al., 2025). Thus, the development of low-cost spectral detectors for component-specific chlorophyll measurement requires the following considerations: (1) employing a small number of sensitive and reference spectral bands for the target chlorophyll components; (2) defining appropriate channel bandwidths; (3) acquiring spectral signals through low-cost, controllable light-source spectral formation at selected bands; and (4) converting the collected reflectance or transmittance signals into absorption-related spectral variables under *in vivo* leaf conditions.

Although the spectral features of Chla and Chlb strongly overlap in leaf spectra acquired *in vivo*, their individual contributions can still be retrieved from hyperspectral data using statistical and mechanistic inversion approaches. Statistical approaches commonly establish empirical relationships between pigment contents and spectral indices or selected spectral features, including PSSR and PSND (Blackburn, 1998b), as well as REPLE and REPIG (Blackburn, 1998a; Wang et al., 2009). More recent data-driven studies have further improved hyperspectral chlorophyll estimation by incorporating phenological information, feature selection, derivative spectral transformations, and machine-learning regressors (Guo et al., 2024). These statistical and data-driven approaches still rely largely on hyperspectral data and high spectral resolution, which limits their direct transfer to low-cost detector design. In mechanistic approaches, radiative transfer models related to Beer–Lambert absorption theory have been used to extract pigment absorption characteristics over broad spectral ranges, typically 400–800 nm at approximately 3 nm resolution (Zhang et al., 2017). These models explicitly account for spectral overlap among pigments and provide physically interpretable inversion results. However, two key limitations remain for practical detector development: (1) their dependence on hyperspectral data and (2) the requirement for a large number of spectral bands, which limits the development of detectors based on controlled light-source spectral formation at only a few selected bands. For detector development, the key is to retain the absorption-additivity concept of Beer–Lambert theory in a simplified and empirically calibrated form while reducing the spectral input to a small set of sensitive and reference bands. In this context, a pseudo-absorption framework with a reference band for optical path length normalization provides a practical basis for spectral overlap separation and band selection under *in vivo* leaf conditions.

The selection of spectral parameters for leaf chlorophyll detectors is commonly derived from full-spectrum hyperspectral datasets. However, excessively high spectral resolution does not necessarily lead to improved performance in low-cost detector development. The main characteristic ranges of Chla and Chlb absorption peaks in leaf spectra acquired *in vivo* are broader than 20 nm (Zhang et al., 2020). Therefore, spectral consistency analysis based on the resampling of hyperspectral data at different spectral resolutions can be used to determine reasonable spectral bandwidths for low-cost detector development (Wang et al., 2022). Commonly, the raw data collected from different channels corresponding to several sensitive and reference spectral bands are obtained in transmission or reflection modes in the developed detector. According to the requirements for detecting target component contents in leaves, these raw spectral data should be transformed into a pseudo-absorption spectral form. Whether leaf spectra are obtained in transmission or reflection form, they are essentially a comprehensive response of the absorption characteristics of various substances in the leaves. Moreover, pseudo-absorption spectra can be generated by taking the logarithm of the reciprocal of reflectance or transmittance [log(1/R) or log(1/T)] (Yoder and Pettigrew-Crosby, 1995), which maps reflectance or transmittance values to an expanded numerical range and enhances absorption features (Blackburn, 1999). Thus, after selecting spectral parameters from full-spectrum hyperspectral data, spectral consistency analysis and pseudo-absorption spectral transformation provide a basis for CabD development.

To develop a leaf Chla and Chlb content detector (CabD), this study collected leaf optical properties, including hyperspectral reflectance data and a wide range of Chla and Chlb contents. A Beer–Lambert-inspired pseudo-absorption linearization framework for effective separation of *in vivo* Chla and Chlb spectral responses, hereafter referred to as BL-OS, was formulated. In this framework, a reference band is introduced for optical path length normalization, which helps reduce the influence of leaf structural variation and separate the overlapping absorption responses of the two pigments. Multi-scale resampling of hyperspectral data and pseudo-absorption spectral transformation were then employed to determine the key parameters for the CabD under development, including the optimal spectral bandwidth configuration. Subsequently, controlled light-source spectral formation at selected wavelengths was combined with photoelectric detection to realize low-cost spectral data acquisition. The resulting CabD was designed to enable direct, *in situ*, non-destructive estimation of leaf Chla and Chlb contents, thereby supporting component-specific pigment information acquisition under *in vivo* leaf conditions.

## 2 Materials and methods

### 2.1 Experimental setup

In order to develop an algorithm for separating the overlapping spectral features of Chla and Chlb from *in vivo* leaf spectra and to verify the accuracy of the developed spectral detector, two experimental procedures were performed: (1) collection of leaf optical properties and (2) validation of the developed CabD device. The instruments used, number of leaf samples, data types, and applications are summarized in **Table 1**.

**Table 1:** Experimental datasets utilized for the development of the spectral overlap separation algorithm and the performance evaluation of the portable CabD device.

Experiment	Instruments	Number of leaf samples	Data type	Application
Leaf optical property collection	ASD FieldSpec 4	236	hyperspectral reflectance data	Developing an algorithm for separating overlapping spectra
	UV-3600 Plus	236	Chla and Chlb Content	
The developed CabD verification	CabD	162	multi-channel LED-source spectra	Calibrating and verifying the developed CabD
	UV-3600 Plus	162	Chla and Chlb Content	
CabD *in situ* validation	CabD	84	multi-channel LED-source spectra	Independent *in situ* validation
	UV-3600 Plus	84	Chla and Chlb contents	

A total of 245 leaf samples were collected for the BL-OS algorithm development phase, and an additional 162 leaf samples were collected for CabD-specific model re-calibration and device verification. A further 84 intact leaf samples were collected for independent *in situ* validation under attached-leaf measurement conditions, resulting in a total of 491 leaf samples. The samples were canopy leaves collected from ten broadleaf species or species groups, including representative taxa such as holly (*Ilex* spp.), sweet osmanthus (*Osmanthus fragrans*), citron (*Citrus medica*), tea tree (*Camellia sinensis*), camellia (*Camellia* spp.), and magnolia (Magnolia denudata). Leaves were selected across various developmental stages—young, mature, and senescent—to ensure representation of lifecycle diversity, and the complete species information, plant type, sampling position, and leaf stage are provided in **Supplementary Table 1**.

### 2.2 Plant leaf data collection

#### 2.2.1 Leaf hyperspectral reflectance data

To minimize pigment degradation and metabolic fluctuations during prolonged *ex situ* handling prior to leaf hyperspectral reflectance data acquisition, green leaves without mechanical damage and exhibiting normal coloration across different developmental stages were selected as samples. Among these samples, the leaves used for *in situ* validation of the CabD device were measured with the CabD device before detachment. Leaf sampling was restricted to 09:00-11:00 h daily to reduce the influence of diurnal irradiance and transpiration variability on pigment composition. Immediately after sampling, leaves were sealed in plastic bags, stored in an ice box to maintain an internal temperature below 4 °C, and swiftly transported to the laboratory. Spectral and biochemical measurements were completed within two hours of collection to minimize further metabolic activity and pigment degradation (Lichtenthaler, 1987).

**Figure 1:**
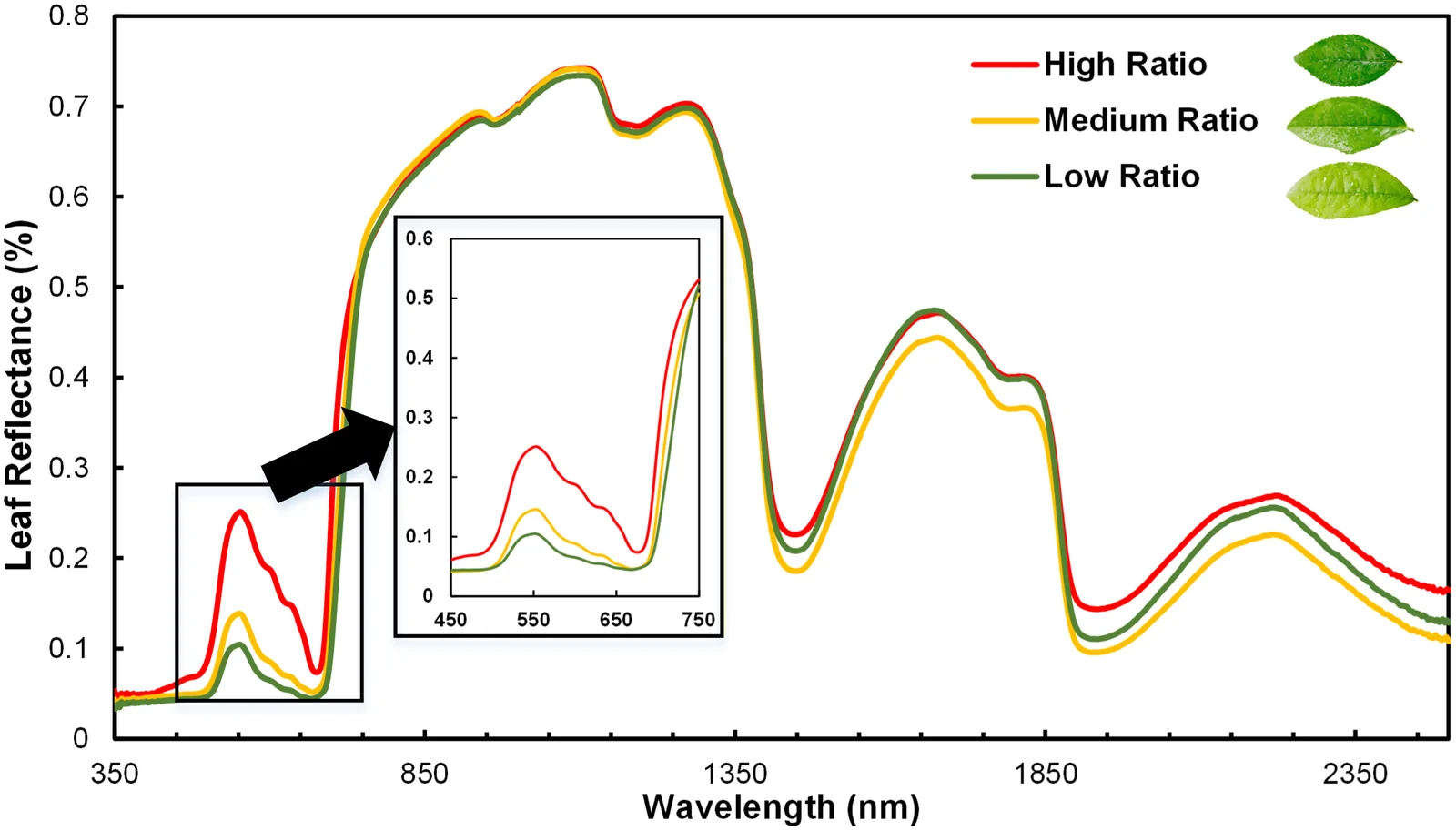
Leaf reflectance spectra of three representative individual leaf samples with high, medium, and low Chla/Chlb ratios. The ratios corresponded to high 3.424 (Chla = 13.756 μg·cm^-2^, Chlb = 4.018 μg·cm^-2^), medium 2.667 (Chla = 24.119 μg·cm^-2^, Chlb = 9.045 μg·cm^-2^), and low 1.911 (Chla = 27.927 μg·cm^-2^, Chlb = 14.613 μg·cm^-2^), respectively.

After the samples had equilibrated to room temperature, spectral measurements were performed in the campus laboratory using a spectral observation platform equipped with an ASD FieldSpec 4 spectroradiometer. To eliminate spectral variability arising from leaf curvature, orientation deviations, and background interference, a quartz plate with high transmittance (≥99%) was used to flatten the leaf surface, thereby ensuring a uniform measurement geometry. Illumination was provided by a halogen lamp at a 45° incident angle, and the fiber-optic probe was positioned vertically above the sample. Before leaf spectral acquisition, background and reference measurements were recorded for radiometric correction. Leaf hyperspectral signals were then collected under the same measurement geometry, and the raw spectral data were converted into calibrated leaf reflectance according to Equation 1 (Zhang et al., 2012):

(1) $$R=R_{s}\times \frac{R_{1}-R_{0}}{1-R_{0}}$$

where R is the calibrated leaf reflectance after instrument correction; *R_s_* denotes the intrinsic calibration coefficient of the spectrometer; *R*_1_ represents the raw measured reflectance; and $R_{0}$ accounts for the ambient background reflectance. Representative calibrated reflectance spectra from three individual leaf samples with high, medium, and low Chla/Chlb ratios of 3.424, 2.667, and 1.911, respectively, are presented in **Figure 1** to illustrate the spectral differences associated with variations in chlorophyll composition. The Chla/Chlb ratio was used as the basis for sample selection because it reflects changes in chlorophyll allocation associated with light adaptation and leaf developmental status (Lichtenthaler, 1987; Taiz et al., 2015).

#### 2.2.2 Measurement of leaf Chla and Chlb content

Immediately following the acquisition of hyperspectral reflectance data, biochemical component measurements were performed to ensure synchronization and consistency between spectral information and physicochemical data. The contents of Chla and Chlb were determined using the classical spectrophotometric method proposed by Lichtenthaler (Lichtenthaler and Wellburn, 1983). Leaf pigments were extracted using a methanol–ethanol mixed solution, and Chla and Chlb contents were measured with a UV-3600 Plus spectrophotometer (Shimadzu, Japan).

#### 2.2.3 Leaf data preprocessing and outlier removal

**Figure 2:**
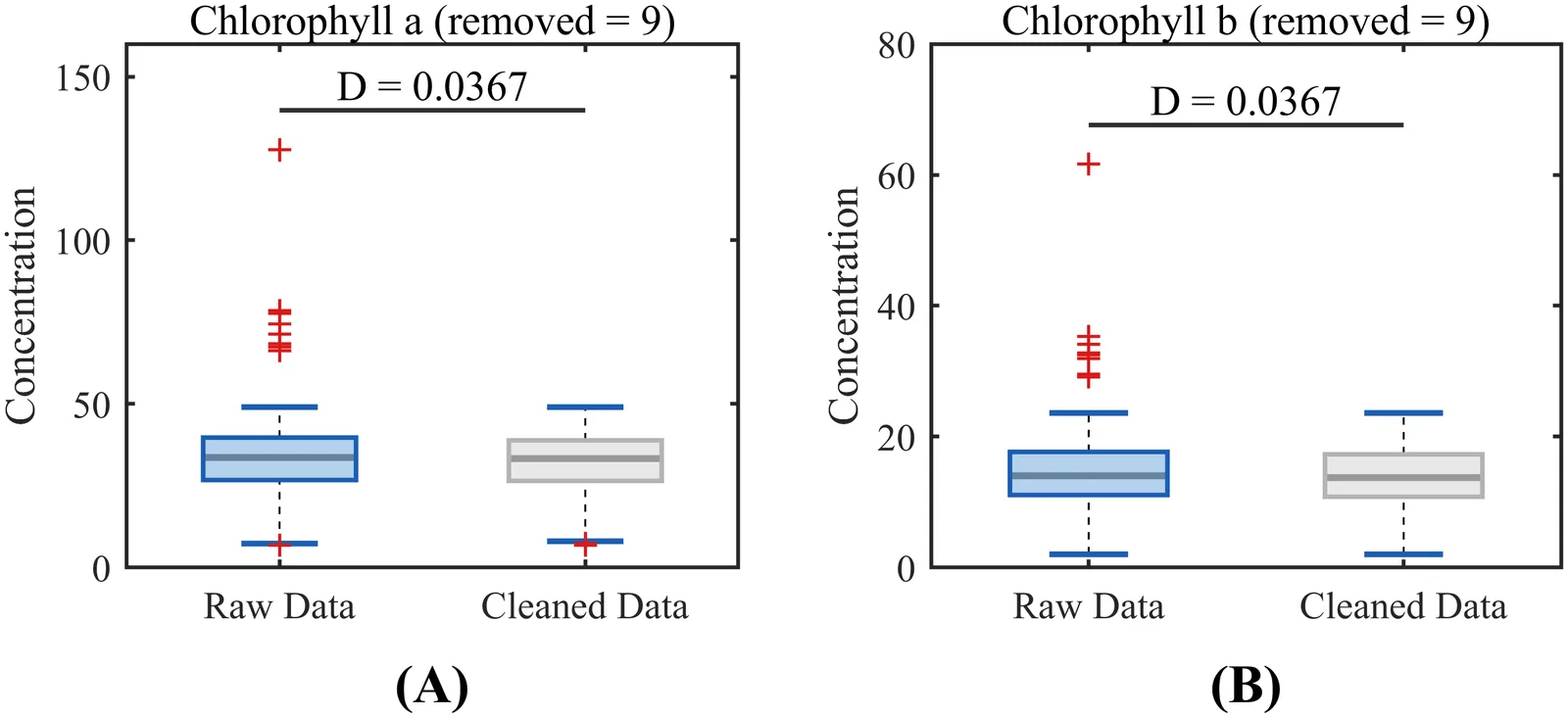
Effects of the two-step IQR-MAD preprocessing procedure on the distributions of Chla and Chlb. **(A)** Chla; **(B)** Chlb. The KS statistic (D) represents the maximum difference between the raw (n = 245) and cleaned (n = 236) data distributions. Outliers are represented by cross symbols (+).

To mitigate potential biases introduced by extreme observations in subsequent model inversion, the Chla and Chlb contents of leaf samples were preprocessed using a two-step outlier detection procedure. First, data points falling outside the interquartile range (IQR) whiskers (1.5 × IQR) were flagged (Krzywinski and Altman, 2014); subsequently, the median absolute deviation (MAD) method with a modified Z-score threshold of 3.5 was applied to identify residual outliers (Rousseeuw and Hubert, 2018). A total of 9 outliers were detected among the initial 245 samples, and 236 valid samples were retained. The excluded observations fell in the upper tails of the Chla and/or Chlb distributions (**Figure 2**), representing statistically extreme chlorophyll values. They came from fully expanded mature leaves sampled in September from holly, tea tree, and camellia. To evaluate whether outlier removal caused a significant shift in the overall data distribution, the raw and cleaned datasets were statistically compared using two-sample Kolmogorov–Smirnov tests. The results showed no statistically significant distributional difference for either Chla (D = 0.0367, p = 0.9963) or Chlb (D = 0.0367, p = 0.9963), indicating that the removal of these outliers did not systematically alter the distribution of the data. Following this preprocessing step, the influence of extreme observations was reduced, thereby providing a more robust input basis for parameter estimation in the subsequent chlorophyll content inversion models. The summary statistics of the preprocessed dataset (n = 236) are presented in **Table 2**. For subsequent BL-OS model development, these preprocessed samples were divided into 157 samples for model fitting and 79 samples for validation.

**Table 2:** Summary statistics of Chla and Chlb contents in the preprocessed datasets used for BL-OS and CabD model development and validation.

Experiment	Type	Max Value (μg·cm^-2^)	Min Value (μg·cm^-2^)	Mean Value (μg·cm^-2^)	Standard deviation (μg·cm^-2^)
Leaf optical property collection	Chla	49.010	6.816	31.762	9.450
	Chlb	23.611	2.008	13.717	4.638
The developed CabD verification	Chla	42.193	14.444	28.330	6.234
	Chlb	14.844	4.914	10.262	1.993
CabD *in situ* validation	Chla	49.003	9.410	29.127	8.568
	Chlb	21.254	4.169	12.927	3.846

### 2.3 Algorithm development and parameter optimization

**Figure 3:**
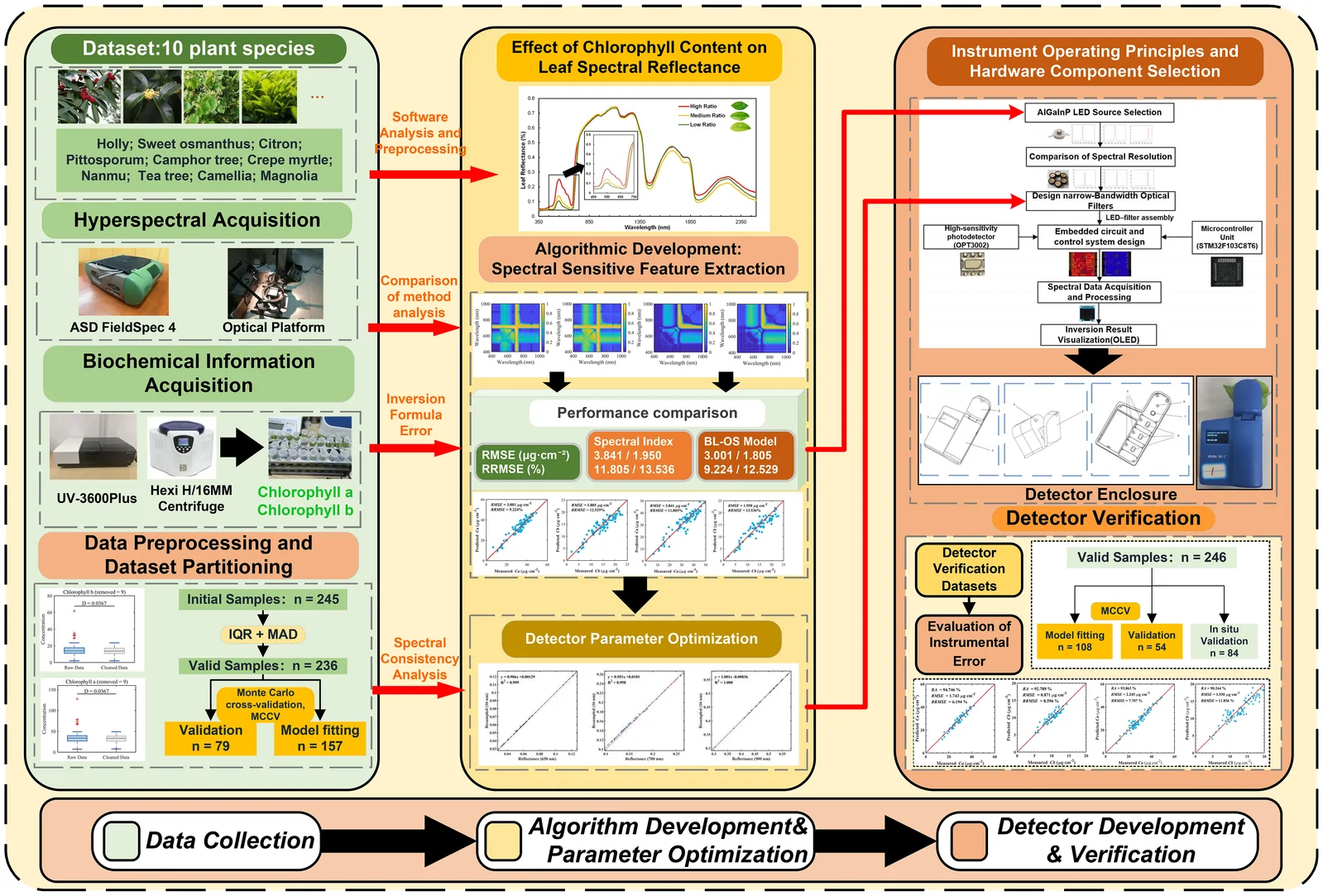
Technical workflow of CabD development, including data collection, algorithm development and parameter optimization, and CabD development and verification.

A systematic workflow for developing the CabD device is illustrated in **Figure 3**, comprising three components: data collection, algorithm development and parameter optimization, and CabD development and verification.

1. A leaf optical property dataset was acquired to support BL-OS algorithm development and spectral parameter optimization, while a separate CabD dataset was collected for device-specific model recalibration and verification, as described in Section 2.2.
2. The pseudo-absorption concept and the absorption-additivity concept of the Beer–Lambert law were combined to develop an empirically calibrated spectral-response overlap separation algorithm and to identify the sensitive and reference central bands for *in vivo* leaf Chla and Chlb using the leaf optical property dataset. Spectral resampling was subsequently employed to optimize the bandwidths of these sensitive and reference bands for integration into the CabD.
3. CabD development and verification: Multi-channel reflectance spectra were acquired using a “spectrum-forming mode” in which narrow-band LED light sources with optimized channel bandwidths centered at the selected wavelengths were sequentially activated. Specific-wavelength LEDs, an OPT3002 photosensitive sensor, and an STM32 microcontroller were integrated into a self-designed circuit to perform leaf reflectance sensing, light source control, and on-board data processing. The CabD was packaged as a portable handheld detector, and its performance was verified using the independent validation dataset.

#### 2.3.1 Development of the spectral overlap effective separation algorithm for *in vivo* broadleaf spectra

In the quantifying analysis in organic solution by Beer-Lambert law, its medium was regarded as a liquid-phase and homogeneous medium (Beer, 1852). Fortunately, the water content was generally more than 50% in the fresh leaves (Wang et al., 2021), and the *in vivo* broadleaf, as a medium for the reflectance or transmittance of the whole leaf, was also regarded as a homogeneous medium (Feret et al., 2008). Thus, at the molecular response scale, the absorbance of Chla and Chlb at their overlapping wavelengths was expressed as a linear relationship, inspired by Beer–Lambert law. This provided a basis for the subsequent pseudo-absorption modeling of *in vivo* broadleaf spectral responses.

(2) $$A_{a,i}=C_{a}\gamma_{a,i}+C_{b}\gamma_{b,i}$$

(3) $$A_{b,j}=C_{a}\gamma_{a,j}+C_{b}\gamma_{b,j}$$

where i and j are the sensitive wavelengths of Chla and Chlb content in organic solution, respectively; $A_{a,i}$ and $A_{b,j}$ are the absorbance values of Chla and Chlb at wavelengths *i* and *j*, respectively; $C_{a}$ and $C_{b}$ are leaf Chla and Chlb contents, respectively; and $\gamma_{a,i}$, $\gamma_{a,j}$, $\gamma_{b,i}$ and $\gamma_{b,j}$ are the absorption coefficients of Chla and Chlb at wavelengths *i* and *j*, respectively. These absorption coefficients ( $\gamma_{a,i}$ and $\gamma_{a,j}$, $\gamma_{b,i}$ and $\gamma_{b,j}$) can characterize and separate the overlapping spectral characteristics of Chla and Chlb in the specific organic solution (Lichtenthaler, 1987).

The spectra of *in vivo* leaves measured by a spectrometer are commonly expressed as reflectance or transmittance (Jacquemoud and Baret, 1990). Because Equations 2, 3 are formulated in terms of absorbance, their application to *in vivo* leaf spectra requires transforming the measured reflectance or transmittance into an absorption spectrum or an analogous absorption spectrum. Accordingly, leaf reflectance *in vivo* can be transformed into an analogous absorption spectrum using the pseudo-absorption approach (Blackburn, 1998b). The corrected pseudo-absorption coefficient $P_{i}$ for an *in vivo* leaf is given by Equation 4.

(4) $$P_{i}=-\text{ln }(R_{i}/R_{N})$$

where $R_{i}$ is the leaf reflectance at wavelength *i*; $R_{N}$ is the leaf reflectance in the near-infrared region that indirectly reflects the optical path length of light–leaf interaction (Sims and Gamon, 2002); and $R_{i}/R_{N}$ represents the leaf relative reflectance after normalizing for optical path length. Thus, Equation 4 yields an analogous absorption spectrum following calibration of the optical path length.

In this study, the water-phase medium assumption for *in vivo* leaf was used only as an approximate basis for transforming solution absorbance into leaf pseudo-absorption. However, Equations 2, 3 were derived for organic solution. Consequently, a transformation from absorbance ( $A_{a,i}$ and $A_{b,j}$) in organic solution to the corrected pseudo-absorption coefficient ( $P_{a,i}$ and $P_{b,j}$) for *in vivo* leaves was required. Based on the linear relationship inspired by the Beer–Lambert law, the transformation coefficients ( $k_{a,i}$, $k_{b,i}$, $\;k_{a,j}$, $k_{b,j},\;l_{i}$, and $l_{j}$) were introduced in linear equations. Furthermore, to relate the absorption performances of Chla and Chlb in organic solution to the pseudo-absorption responses of *in vivo* leaves, empirical transformation coefficients were introduced in the linearized formulation. Therefore, the pseudo-absorption responses of Chla and Chlb *in vivo* are expressed by the following equations.

(5) $$P_{a,i}=k_{a,i}C_{a}\gamma_{a,i}+k_{b,i}C_{b}\gamma_{b,i}+l_{i}$$

(6) $$P_{b,j}=k_{a,j}C_{a}\gamma_{a,j}+k_{b,j}C_{b}\gamma_{b,j}+l_{j}$$

where $P_{a,i}$ and $P_{b,j}$ are the pseudo-absorption responses of Chla and Chlb at wavelengths *i* and *j*.

Since the absorption terms ( $\gamma_{a,i}$ and $\gamma_{a,j}$, $\gamma_{b,i}$, and $\gamma_{b,j}$) are related to the absorption-additivity relationship of Beer–Lambert theory, Equations 5, 6 can be used to represent the overlapping absorption-related responses of Chla and Chlb in an empirical linearized form. The contents of Chla and Chlb *in vivo* leaf can therefore be expressed by the following equations (Equations 7, 8), which describe the relationship between pigment contents and pseudo-absorption variables in the BL-OS framework:

(7) $$C_{a}=\frac{P_{b,j}\times k_{b,i}\gamma_{b,i}-P_{a,i}k_{b,j}\gamma_{b,j}-(l_{j}\times k_{b,i}\gamma_{b,i}-l_{i}k_{b,j}\gamma_{b,j})}{k_{a,j}\gamma_{a,j}k_{b,i}\gamma_{b,i}-k_{a,i}\gamma_{a,i}k_{b,j}\gamma_{b,j}}$$

(8) $$C_{b}=\frac{P_{a,i}\times k_{a,j}\gamma_{a,j}-P_{b,j}k_{a,i}\gamma_{a,i}-(l_{i}\times k_{a,j}\gamma_{a,j}-l_{j}k_{a,i}\gamma_{a,i})}{k_{a,j}\gamma_{a,j}k_{b,i}\gamma_{b,i}-k_{a,i}\gamma_{a,i}k_{b,j}\gamma_{b,j}}$$

Given that the transformation coefficients ( $k_{a,i}$, $k_{b,i}$, $\;k_{a,j}$, $k_{b,j}$, $l_{i}$, and $l_{j}$) were treated as empirical transformation terms, and the absorption coefficients ( $\gamma_{a,i}$, $\gamma_{b,i}$, $\;\gamma_{a,j}$, and $\gamma_{b,j}$) are fixed at the selected wavelengths, the combined effects of these absorption coefficients and empirical transformation coefficients were incorporated into fitted effective coefficients ( $K_{a,i}$, $K_{b,i}$, $\;K_{a,j}$, $K_{b,j}$, $L_{i}$ and $L_{j}$) to simplify Equations 7, 8. By combining these simplified equations with Equation 4, the inversion models for *in vivo* Chla and Chlb content are described by the following equations (Equations 9, 10):

(9) $$C_{a}=K_{a,i}\times ln(\frac{R_{N}}{R_{i}})-K_{b,i}\times ln(\frac{R_{N}}{R_{j}})-L_{i}$$

(10) $$C_{b}=K_{b,j}\times ln(\frac{R_{N}}{R_{j}})-K_{a,j}\times ln(\frac{R_{N}}{R_{i}})-L_{j}$$

where $R_{i}$, $R_{j}$ and $R_{N}$ represent the reflectance at the sensitive wavelength of Chla, the sensitive wavelength of Chlb, and the near-infrared reference wavelength, respectively.

Because the model parameters ( $K_{a,i}$, $K_{b,i}$, $\;K_{a,j}$, $K_{b,j}$, and $L_{i}$ and $L_{j}$) in Equations 9, 10 incorporate the combined effects of the absorption coefficients ( $\gamma_{a,i}$ and $\gamma_{a,j}$, $\gamma_{b,i}$ and $\gamma_{b,j}$) of Chla and Chlb and the empirical transformation coefficients ( $k_{a,i}$, $k_{b,i}$, $\;k_{a,j}$, $k_{b,j},\;l_{i}$, and $l_{j}$), their values were determined by minimum-distance fitting between predicted and measured contents of Chla and Chlb *in vivo* based on the measured leaf reflectance.

#### 2.3.2 Parameter optimization of the developed detector

The CabD device developed in this study is intended as a multi-channel spectral detector for determining Chla and Chlb contents *in vivo* leaf. Accordingly, parameter optimization was performed prior to hardware implementation, including sensitive wavelength selection and spectral channel bandwidth optimization.

##### 2.3.2.1 Sensitive spectrum wavelength selection

According to Equations 9 and 10, the detection wavelengths required by the CabD for estimating Chla and Chlb contents *in vivo* consist of the sensitive wavelengths *i* and *j* for Chla and Chlb, respectively, together with a near-infrared wavelength for normalizing optical path length.

Using the evaluation metrics R^2^ and RMSE, the wavelengths and model parameters ( $K_{a,i}$, $K_{b,i}$, $\;K_{a,j}$, $K_{b,j}$ and $L_{i}$ and $L_{j}$) in Equations 9, 10 were fitted by minimizing the distance between the measured and predicted contents of leaf Chla and Chlb. To assess the stability of the BL-OS model, repeated Monte Carlo cross-validation was conducted according to a previously established method (Xu and Liang, 2001), using 157 of the 236 samples for calibration and the remaining 79 samples for validation in each partition. Model coefficients were re-estimated for each partition, and RMSE and RRMSE were calculated for Chla and Chlb. The calibration and validation performance distributions are presented in **Supplementary Table 3**, and the validation RMSE distributions are shown in **Supplementary Figure 1**. For comparison, conventional wavelength selection methods based on spectral indices including the selected RI (Chappelle et al., 1992), DI (Tucker, 1979), NDI (Gitelson and Merzlyak, 1994), and RDI (Roujean and Breon, 1995) were also employed to evaluate the performance of the proposed wavelength selection approach.

##### 2.3.2.2 Spectral channel bandwidth optimization

In this study, the CabD was developed to enable convenient, low-cost, portable, and non-destructive acquisition of Chla and Chlb contents from plant leaves under *in vivo* conditions. However, the selected spectral parameters (sensitive and near-infrared wavelengths) derived from leaf hyperspectral data possess high spectral resolution, which is associated with higher hardware cost. Therefore, spectral channel bandwidth optimization of these selected parameters was necessary to reduce detector cost while maintaining acceptable accuracy.

The spectral characteristics of both Chla and Chlb *in vivo* exhibit broadband absorption features (Curran, 1989). In this study, spectral resampling was employed to optimize the spectral channel bandwidth of the CabD. Based on the original leaf hyperspectral data with 3 nm spectral resolution from the “Leaf optical property collection” dataset, the new reflectance spectra centered at 650, 700, and 900 nm were resampled using the method of Yang (Yang, 2022), each with bandwidths of 6, 10, 16, and 30 nm. Specifically, the reflectance associated with each original spectral interval was weighted according to its overlap width with the target spectral interval, and the weighted sum was normalized by the total overlap width.

The candidate channel bandwidth range for the selected spectral parameters of the CabD was determined by evaluating the spectral consistency between the original 3 nm reflectance spectra and the spectra resampled to different bandwidths using R² and RMSE, while also taking into account the constraints of low-cost hardware development.

#### 2.3.3 CabD design and development

**Figure 4:**
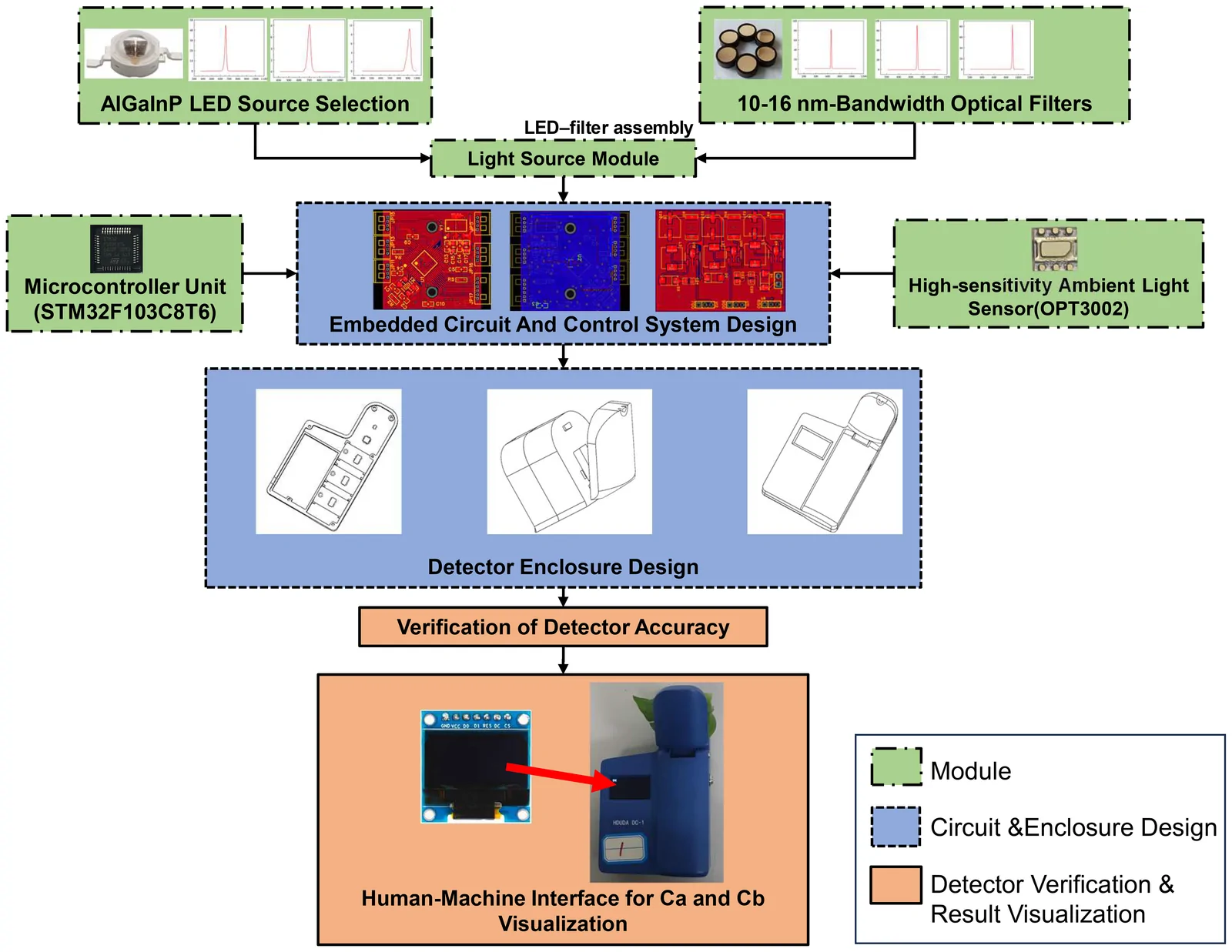
Workflow of CabD design and development.

Based on the sensitive and reference bands for Chla and Chlb identified using the BL-OS method in Section 2.3.2, this study employed several discrete sensitive bands instead of traditional continuous hyperspectral sampling to achieve high-accuracy retrieval of chlorophyll content. Unlike conventional hyperspectral instruments, which obtain continuous spectral information through prism- or grating-based spectral dispersion, this study adopted a light-source-controlled spectral acquisition strategy. Specifically, target-band spectral signals were generated using a combination of LEDs and narrow-band optical filters (Lopin et al., 2025). This design reduces the dependence on complex spectral splitting structures and high-cost detection modules, thereby providing a technical basis for the low-cost design and development of the CabD instrument. The workflow of the CabD system is illustrated in **Figure 4**, and the main components together with their estimated costs are summarized in **Supplementary Table 2**. The detector mainly consists of three modules: a spectral detection module, a control and processing module, and a mechanical housing module.

##### 2.3.3.1 Light source module

In contrast to spectral collection based on prism splitting, a mode that controls the spectral characteristics of the irradiation light source offers a lower-cost detector development pathway (Gitelson et al., 2003). Accordingly, the spectral detector module of the CabD was designed such that spectral acquisition is achieved by accurately controlling the spectral characteristics of the irradiation light source in conjunction with a corresponding reflected radiation sensor, thereby significantly reducing the overall development cost.

The irradiation light source of the CabD consists of AlGaInP Light-Emitting Diodes (LEDs) combined with Optical Bandpass Filters (OBFs), with the following constraints imposed on the spectral characteristics: (1) specific central wavelengths of the LEDs were selected according to the sensitive wavelengths of Chla and Chlb and their associated reference wavelength obtained from the sensitive wavelength selection results in Section 2.3.2; and (2) the channel bandwidths of the OBFs were selected according to the results of spectral channel bandwidth optimization in Section 2.3.2. These constraints ensure a stable and spectrally specific incident light source.

The control and data processing subsystem was implemented using a low-power, high-reliability microcontroller unit (MCU, STM32F103C8T6), which drives and regulates the light source module, synchronizes data acquisition, and manages communication with the optical sensor. Acquisition and processing of reflected signals are performed by the embedded control subsystem, whose core functions include light source timing control, sensor data acquisition, and real-time reflectance calculation. Before leaf measurement, the dark DN signal was recorded with all LEDs switched off and the optical chamber closed. A 99% BaSO_4_ white reference was subsequently measured repeatedly for each channel, and the mean dark-current-corrected DN values were stored in the embedded software as fixed white-reference parameters. The spectral reflectance of the leaf at the sensitive and reference wavelengths for Chla and Chlb was calculated within the MCU by dividing the dark-current-corrected leaf DN by the corresponding dark-current-corrected white-reference DN. To evaluate relative deviations in repeated optical measurements, the relative root-mean-square error (RRMSE) was introduced and defined in Equation 11:

(11) $$RRMSE=\sqrt{\frac{1}{n}\sum_{i=1}^{n}\;{\left(\frac{x_{i}-\bar{x}}{\bar{x}}\right)}^{2}}\times 100\;\%$$

where $x_{i}$ is the i-th evaluated value, $\bar{x}$ is the mean value, and *n* is the number of observations.

Consequently, Equations 9, 10, originally derived from hyperspectral data, were adapted to Equations 12, 13 for retrieving Chla and Chlb contents using the CabD:

(12) $$C_{a}=K_{a,i}^{'}\text{ln}(\frac{{\text{DN}}_{\text{N},\text{L},\text{R}}/{\text{DN}}_{\text{N},\text{W},\text{R}}}{{\text{DN}}_{\text{i},\text{L},\text{R}}/{\text{DN}}_{\text{i},\text{W},\text{R}}})-K_{b,i}^{'}\text{ln}(\frac{{\text{DN}}_{\text{N},\text{L},\text{R}}/{\text{DN}}_{\text{N},\text{W},\text{R}}}{{\text{DN}}_{\text{j},\text{L},\text{R}}/{\text{DN}}_{\text{j},\text{W},\text{R}}})-L_{i}^{'}$$

(13) $$C_{b}=K_{b,j}^{'}\text{ln}(\frac{{\text{DN}}_{\text{N},\text{L},\text{R}}/{\text{DN}}_{\text{N},\text{W},\text{R}}}{{\text{DN}}_{\text{j},\text{L},\text{R}}/{\text{DN}}_{\text{j},\text{W},\text{R}}})-K_{a,j}^{'}\text{ln}(\frac{{\text{DN}}_{\text{N},\text{L},\text{R}}/{\text{DN}}_{\text{N},\text{W},\text{R}}}{{\text{DN}}_{\text{i},\text{L},\text{R}}/{\text{DN}}_{\text{i},\text{W},\text{R}}})-L_{j}^{'}$$

where $DN_{N,L,R}$, $DN_{i,L,R}$ and $DN_{j,L,R}$ are the leaf-reflected DN values measured by the OPT3002 sensor at the near-infrared reference wavelength and the sensitive wavelengths i and j, respectively; $DN_{N,W,R}$, $DN_{i,W,R}$ and $DN_{j,W,R}$ are the corresponding DN values measured from the white reference panel.

Due to the differences in spectral resolution, sensor response, dark current, and other factors between the ASD FieldSpec 4 spectroradiometer and the CabD, the parameters ( $K_{a,i}^{'}$, $K_{b,i}^{'}$, $L_{i}^{'}$, $K_{b,j}^{'}$, $K_{a,j}^{'}$ and $L_{j}^{'}$) in Equations 12, 13 are different from the corresponding parameters ( $K_{a,i}$, $K_{b,i}$, $\;K_{a,j}$, $K_{b,j}$ and $L_{i}$ and $L_{j}$) in Equations 9, 10. Therefore, these device-specific parameters should be calibrated.

##### 2.3.3.2 Circuit integration and optical enclosure design

The CabD hardware system is configured with an STM32F103C8T6 microcontroller as the central processing unit, while an AMS1117-3.3V voltage regulator supplies a stable operating voltage to the electronic circuitry. A PT4115 constant-current driver is employed to drive the light source module, and pulse-width modulation (PWM) is adopted to regulate the illumination intensity and synchronize the emission sequence. Leaf reflectance signals are acquired using an OPT3002 sensor via I²C communication, thereby ensuring stable signal acquisition and a favorable signal-to-noise ratio.

With respect to the optical configuration, the excitation light emitted by the LED module irradiates the leaf surface at a fixed incident angle of 45°, whereas the sensor captures the reflected light signal along the surface-normal direction. In contrast to conventional hyperspectral instruments that rely on high-precision dispersive optical assemblies, CabD employs industrial-grade, general-purpose electronic and optoelectronic components, thereby substantially reducing hardware costs. The workflow of the CabD system is illustrated in **Figure 4**, and the main components together with their estimated costs are summarized in **Supplementary Table 2**. The per-unit component cost of a prototype is approximately CNY 830–1590, demonstrating a cost-efficient design strategy oriented toward high-throughput field applications.

**Figure 5:**
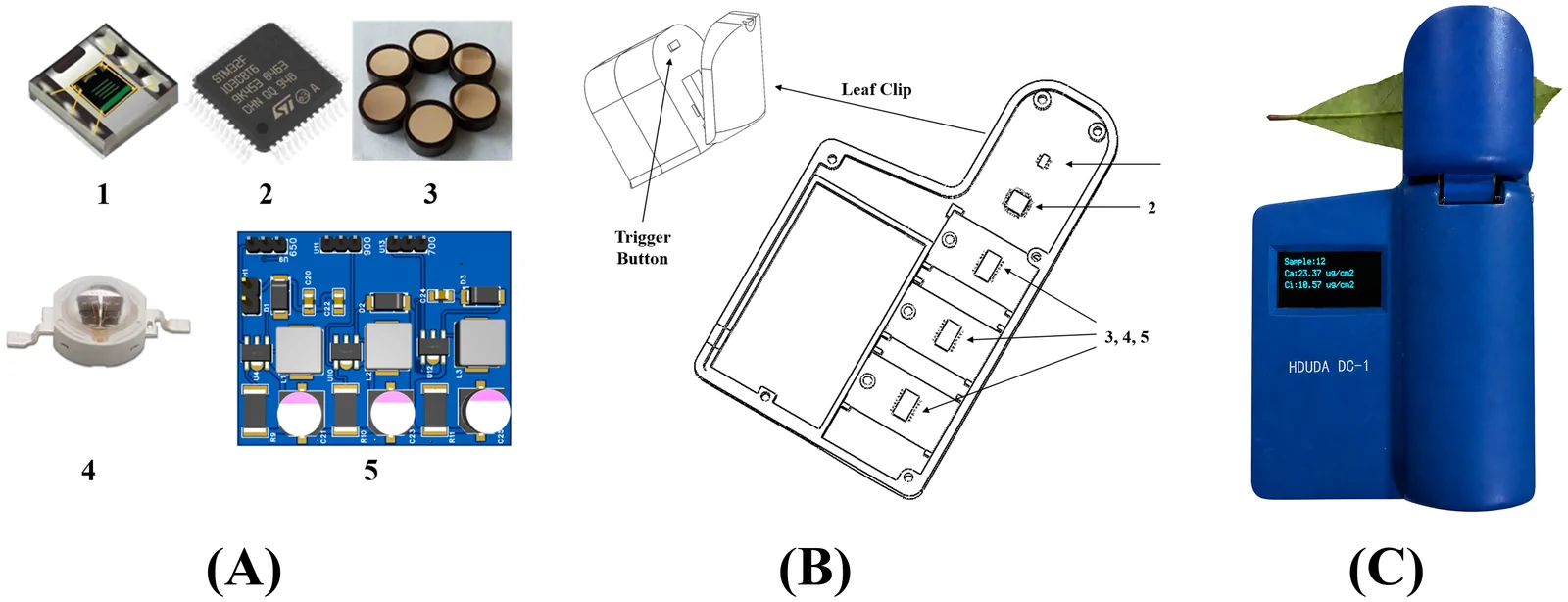
Structure and module composition of the CabD. **(A)** Core internal modules: 1, photoelectric sensor; 2, STM32 microcontroller; 3, narrow-band filters; 4, LED light source; 5, drive circuit board (see **Supplementary Table 2**). **(B)** Main functional module distribution, illustrating the internal layout with a leaf clip and trigger button to ensure a stable measurement environment. **(C)** The assembled CabD prototype (prototype designation: HDUDA DC-1) displaying real-time retrieval results.

To suppress ambient light interference and meet the demands of portable field operation, the device adopts a fully enclosed handheld design with a housing made of integrally molded ABS material with high light-shielding capability. The housing integrates an OLED display for real-time readout of measurement parameters, a measurement trigger button, and an elastic leaf clip. The core components, internal structural layout, and assembled prototype of CabD are presented in **Figure 5**. Specifically, **Figure 5A** shows the core optoelectronic and control modules, **Figure 5B** illustrates the internal arrangement of the leaf clip, trigger button, and functional modules, and **Figure 5C** shows the assembled handheld prototype.

This mechanical structure, together with the associated circuit design, jointly ensures stable illumination conditions and consistent measurement geometry during operation, thereby providing an essential prerequisite for the stable and reliable application of the leaf chlorophyll retrieval models (Equations 12, 13).

##### 2.3.3.3 Software architecture and model validation

The CabD software was developed based on the STM32 HAL library and employs a finite state machine architecture to implement time-division multiplexing control of LED light sources at different wavelengths. Before measurement, an automatic software-controlled LED warm-up period of more than 11 s (t > 11 s) was applied to reduce transient output fluctuations caused by LED thermal effects and ensure stable signal acquisition. To mitigate instantaneous signal fluctuations induced by the thermal effects of the LEDs, a stabilization delay is introduced after each source switching event before valid reflectance data are acquired and calculated. Subsequently, the Chla and Chlb retrieval models, which operate on a spectral overlap separation mechanism, are executed, and the results are displayed in real time on a 0.96-inch OLED screen. The CabD supports two data management modalities: first, data can be stored offline by connecting an external TF card storage module via the UART interface; second, data can be transmitted wirelessly to a host computer or mobile terminal via an ESP8266 Wi-Fi module or an HC-05 Bluetooth module, addressing the requirements of remote monitoring and centralized management. Upon compilation, the software is programmed into the internal Flash memory of the STM32 microcontroller and executes automatically following a power-on reset.

For CabD-specific calibration and validation, an independent CabD dataset containing 162 leaf samples was used. Repeated stratified Monte Carlo cross-validation was conducted according to a previously established method (Xu and Liang, 2001) to evaluate the stability of the CabD-specific retrieval model. In each partition, 108 of the 162 samples were used to recalibrate the device-specific coefficients in the CabD retrieval models, and the remaining 54 samples were used for validation. The calibration and validation performance distributions are presented in **Supplementary Table 3**, and the validation RMSE distributions are shown in **Supplementary Figure 1**. The Chla and Chlb contents determined by standard spectrophotometry served as reference values. The calibration set was used to calibrate the model parameters, and the predictive accuracy was subsequently evaluated on the validation set. Model performance was assessed using root mean square error (RMSE), coefficient of determination (R²), relative root-mean-square error (RRMSE), and relative accuracy (RA). RMSE, R², and RA were calculated according to Equations 14–16, respectively, while RRMSE was calculated as RMSE divided by the mean measured value.

(14) $$RMSE=\sqrt{\frac{1}{n}\sum_{i=1}^{n}{\left(y_{i}-\hat{y_{i}}\right)}^{2}}$$

(15) $$R^{2}=1-\frac{\sum_{i=1}^{n}{\left(y_{i}-\hat{y_{i}}\right)}^{2}}{\sum_{i=1}^{n}{\left(y_{i}-\bar{y}\right)}^{2}}$$

(16) $$RA=(1-\frac{\sum_{i=1}^{n}|y_{i}-\hat{y_{i}}|}{n\cdot \bar{y}})\times 100\%$$

where n is the number of samples in the validation set; $y_{i}$ and $\hat{y_{i}}$ denote the measured and predicted values for the i-th sample, respectively; and $\bar{y}\;$is the mean of the measured values.

To evaluate the operational repeatability of CabD, 12 leaf samples were repeatedly measured at different orientations. Measurements were taken from interveinal regions while avoiding the midrib and major veins, with the midrib oriented either parallel or perpendicular to the longitudinal axis of the measurement window. The illumination and detection geometry remained unchanged. Five measurements were obtained for each sample under different leaf orientations. For each sample, measurement variability was evaluated separately for Chla and Chlb using the maximum, minimum, mean, and RRMSE of the five measurements.

## 3 Results

### 3.1 Selection of spectral parameters for CabD

#### 3.1.1 Selection and analysis of sensitive spectral bands for Chla and Chlb

**Figure 6:**
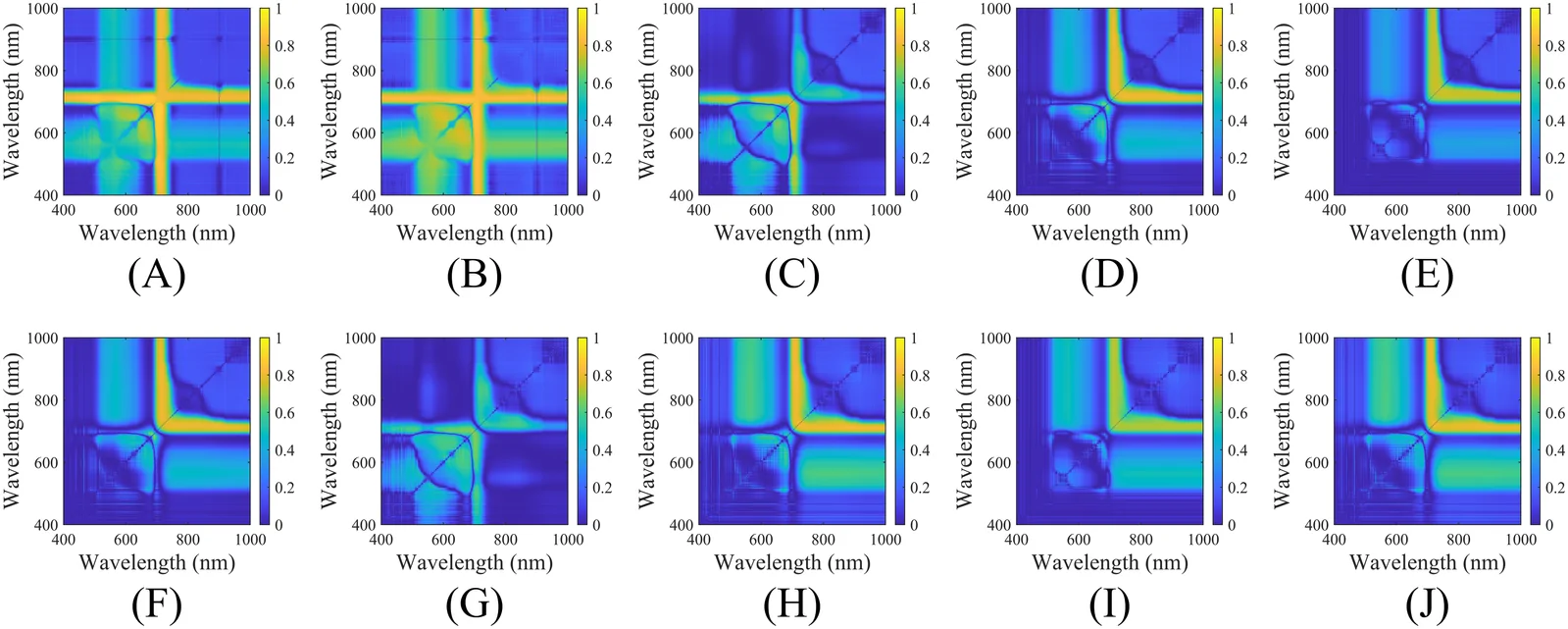
Coefficients of determination (R²) for chlorophyll inversion: **(A, B)** Chla and Chlb using the BL-OS method, respectively; **(C–F)** two-band combinations of DI, NDI, RDI, and RI for Chla, respectively; and **(G–J)** the corresponding two-band combinations for Chlb.

In this section, the BL-OS method (Beer, 1852) was compared with conventional spectral-index methods for selecting sensitive bands and constructing inversion models. Both methods utilized the calibration dataset (n = 157, as described in Section 2) to correlate spectral data (400–1000 nm) with Chla and Chlb contents. The accuracy and stability of the resulting models were subsequently evaluated using the independent validation dataset (n = 79) (results shown in **Figure 6**).

Analysis of the BL-OS method indicated that the absorption intensity of Chla decreased with increasing wavelength within the 700–720 nm range, reaching peak inversion accuracy at 700 nm. Consequently, 700 nm was identified as the optimal sensitive band for Chla (**Figure 6A**). For Chlb, a strong correlation was observed within the 600–660 nm range. To minimize interference from Chla absorption in the red region while leveraging the strong absorption peak of Chlb near 650 nm (Lu et al., 2006), 650 nm was selected as the optimal sensitive band for Chlb (**Figure 6B**). For comparison, **Figures 6C–F** present the coefficients of determination (R^2^) for the two-band combinations of DI, NDI, RDI, and RI for Chla, whereas **Figures 6G–J** present the corresponding results for Chlb. Based on these specific wavelengths, the derived inversion models (Equations 17, 18) were established and evaluated on the validation set.

(17) $$Ca=40.960\;\times \;ln(\frac{R_{900}}{R_{700}})-16.359\;\times \;ln(\frac{R_{900}}{R_{650}})+7.699$$

(18) $$Cb=-4.757\;\times \;ln(\frac{R_{900}}{R_{650}})+17.409\;\times \;ln(\frac{R_{900}}{R_{700}})-1.904$$

where *Ca* and *Cb* denote the contents of Chla and Chlb (μg·cm^-2^), respectively.

**Figure 7:**
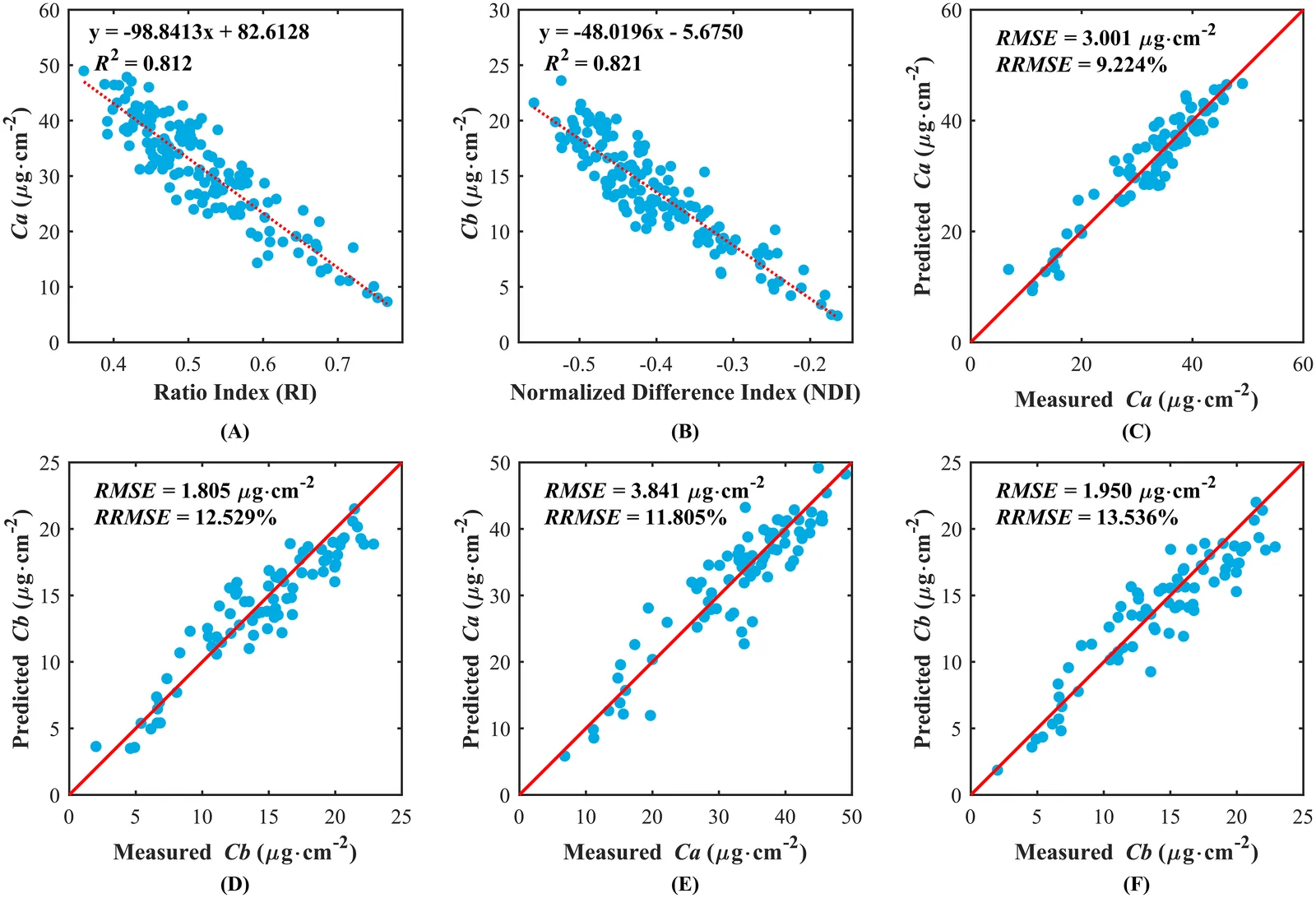
Comparison of the selected conventional spectral-index models and the BL-OS method for chlorophyll retrieval. **(A)** Calibration relationship between the selected RI (R715/R903) and Chla. **(B)** Calibration relationship between the selected NDI [(R711 - R882)/(R711 + R882)] and Chlb. **(C)** Validation results for Chla based on the BL-OS method. **(D)** Validation results for Chlb based on the BL-OS method. **(E)** Validation results of the selected RI model for Chla. **(F)** Validation results of the selected NDI model for Chlb.

The RI for Chla showed the strongest correlation using the 715–903 nm combination, where the linear regression model and R² = 0.812 are presented in **Figure 7A**, whereas the NDI for Chlb peaked at the 711–882 nm combination, with the corresponding model and R² = 0.821 displayed in **Figure 7B**.

To further assess the inversion accuracy, the validation subset was used to compare predicted versus measured chlorophyll contents for both approaches (**Figures 7C–F**). For the conventional spectral-index models, the predicted values were distributed around the 1:1 line, yielding RMSE values of 3.841 μg·cm^-2^ for Chla and 1.950 μg·cm^-2^ for Chlb, with corresponding RRMSE values of 11.805% and 13.536%, respectively. In comparison, the BL-OS method produced predicted versus measured values that were more closely distributed around the 1:1 line, particularly for Chla. The RMSE values for Chla and Chlb were lower at 3.001 μg·cm^-2^ and 1.805 μg·cm^-2^, respectively, while the corresponding RRMSE values decreased to 9.224% and 12.529%. These results indicate that the BL-OS method substantially reduced the absolute prediction error and improved the relative prediction accuracy for Chla, while achieving comparable prediction accuracy for Chlb.

Comparing the two approaches, the spectral index method enhances spectral sensitivity merely through numerical signal amplification. In contrast, the BL-OS method provides a physically interpretable representation by using different absorption-related responses at selected sensitive bands to reduce overlap-related interference. This physically interpretable feature representation reduced prediction errors by approximately 21.9% for Chla and 7.4% for Chlb compared to the selected conventional spectral-index models, indicating a clear improvement for Chla and comparable validation performance for Chlb.

Given its quantitative performance, physical interpretability, and specific band sensitivity in the present dataset, the BL-OS method provides a suitable basis for integration into portable instrumentation. Based on these findings, 650 nm, 700 nm, and 900 nm, with 900 nm serving as the near-infrared reference wavelength, were definitively selected as the core operational wavelengths for the development of the CabD system.

#### 3.1.2 Selection of spectral-channel bandwidth

After designating 650 nm, 700 nm, and 900 nm as the sensitive and reference wavelengths, narrow bandpass filters were applied to constrain the emission bandwidth of the discrete LED sources. This design sought to balance spectral resolution against the signal-to-noise ratio while simultaneously evaluating the influence of broad-spectrum emission on spectral acquisition, thereby enabling the selection of optimal operating wavebands. Consequently, low-cost, high-precision acquisition of wavelength-specific intensity signals was achieved using a broadband photodetector.

Although the spectral resolution realized by this approach is lower than that of the ASD spectroradiometer (approximately 3 nm), previous studies have established that adjacent bands in surface reflectance spectra exhibit strong correlations, and data in the vicinity of characteristic wavebands remain continuous and consistent (Cheng and Guo, 2013). To evaluate the preservation of spectral information after reducing spectral resolution, a spectral consistency analysis was undertaken. Specifically, the original leaf spectra acquired at a 3 nm resolution (see Section 2.2.1) were resampled to bandwidths of 6 nm, 10 nm, 16 nm, and 30 nm for the wavebands pertinent to chlorophyll inversion. The resampling procedure followed the methodology described in (Yang, 2022).

**Figure 8:**
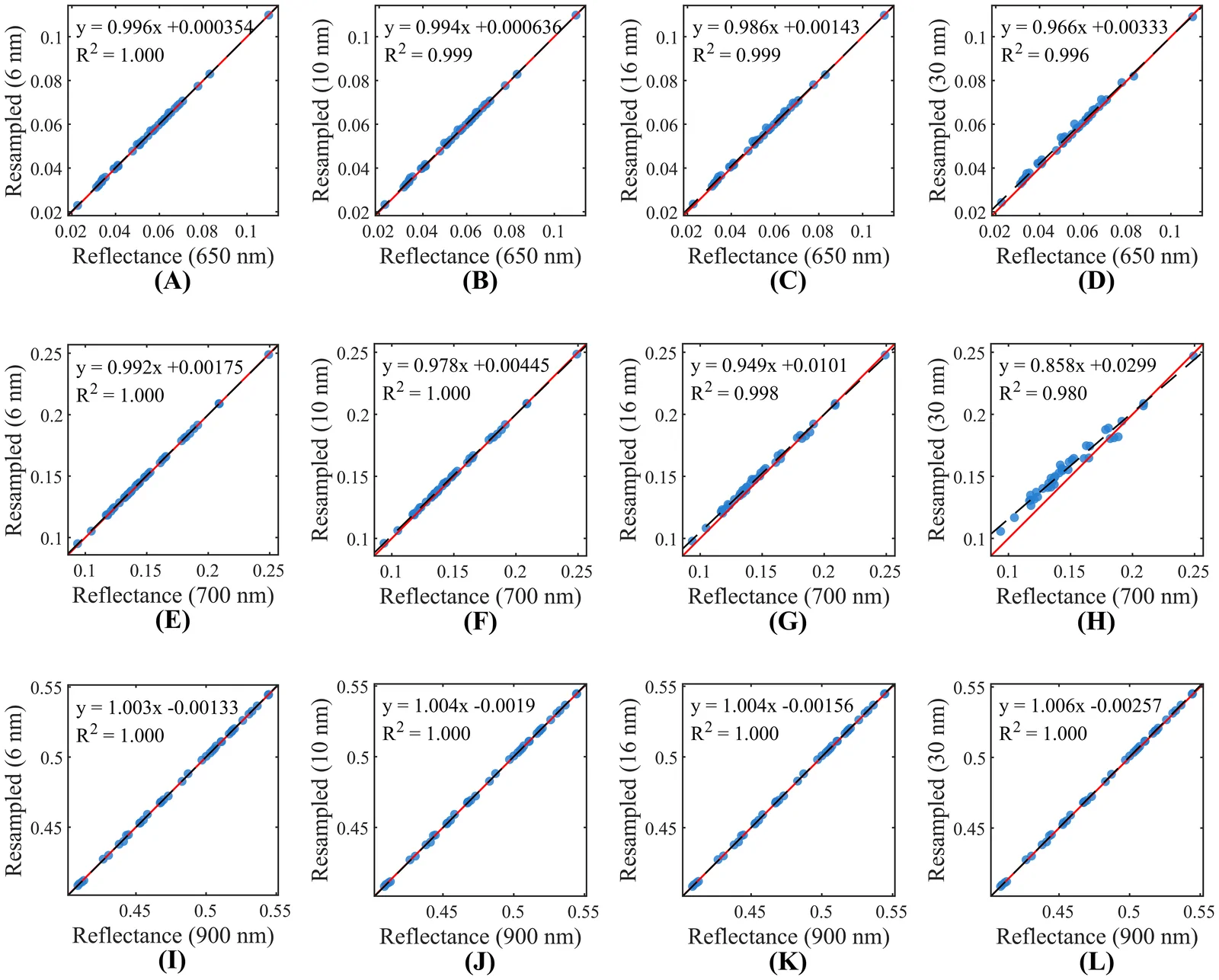
Resampling comparison of reflectance spectra at the three selected wavelengths under different bandwidths. **(A–D)** Reflectance spectra centered at 650 nm with bandwidths of 6, 10, 16, and 30 nm, respectively. **(E–H)** Reflectance spectra centered at 700 nm with bandwidths of 6, 10, 16, and 30 nm, respectively. **(I–L)** Reflectance spectra centered at 900 nm with bandwidths of 6, 10, 16, and 30 nm, respectively.

**Figure 9:**
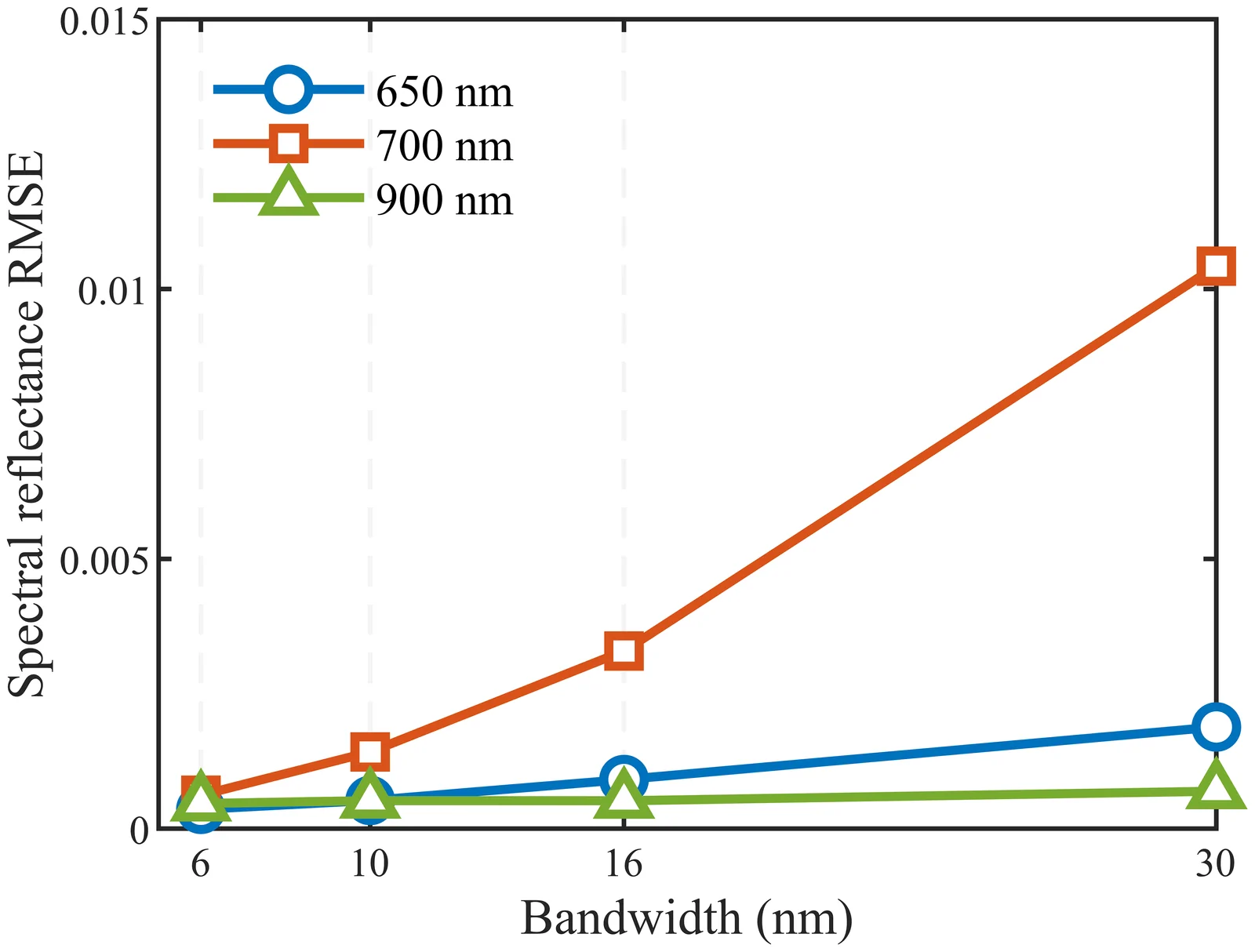
Reflectance resampling RMSE between different spectral bandwidths and the original spectral resolution at 650 nm, 700 nm, and 900 nm. The gray dashed lines indicate different bandwidth positions.

The spectral consistency analysis (**Figure 8**) shows that the measured leaf reflectance spectra at 650 nm, 700 nm, and 900 nm are in excellent agreement with the resampled data at different bandwidths, with the R² approaching 1 at all three wavelengths. **Figure 9** further reveals the influence of bandwidth on reflectance resampling error: When the bandwidth increases from 16 to 30 nm, the reflectance RMSE rises sharply at 700 nm, more moderately at 650 nm, and only slightly at 900 nm; when the bandwidth is below 6 nm, spectral consistency barely improves, whereas the instrument cost rises significantly. Thus, the 6–16 nm bandwidth range maintains good spectral consistency while keeping the reflectance resampling error at a relatively low level.

**Figure 10:**
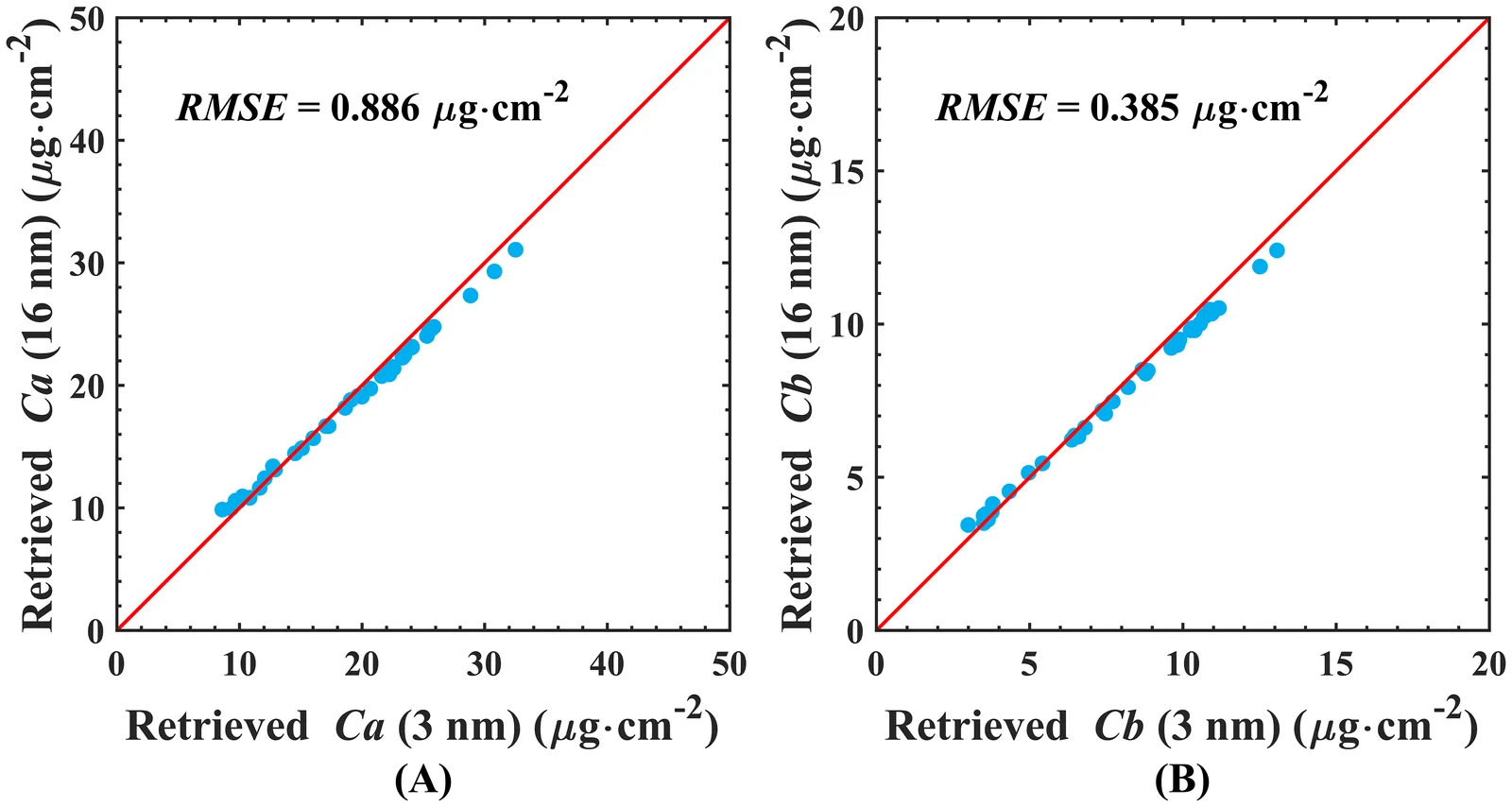
Comparison of chlorophyll inversion RMSE between the original 3 nm hyperspectral data and the 16 nm resampled data. **(A)** Chla; **(B)** Chlb.

To examine the fidelity of spectral information within this range, the maximum bandwidth of 16 nm was selected for resampling, and the retrieval results were compared with those derived from the original 3 nm hyperspectral data (**Figure 10**). Validation using 40 samples from the leaf optical properties dataset demonstrates that the RMSE values of Chla and Chlb obtained from the 16 nm resampled data are 0.886 μg·cm^-2^ and 0.385 μg·cm^-2^, respectively, which are comparable to those from the original 3 nm data, indicating that the 16 nm resampled spectra preserve the core spectral information required for chlorophyll retrieval.

Integrating the results of spectral consistency analysis, reflectance resampling error analysis, and resampling fidelity verification, this study identifies 6–16 nm as a feasible bandwidth range for CabD filter selection. This bandwidth range balances spectral information retention and cost, providing a direct basis for the subsequent selection of instrument filters. The excellent spectral consistency also supports the use of filter-based spectral acquisition within this bandwidth range, laying a technical foundation for prototype development.

### 3.2 Model development and validation of the CabD for non-destructive Chla and Chlb detection

#### 3.2.1 Light source calibration with controlled spectral formation

##### 3.2.1.1 Spectral performance assessment of the CabD integrated light source

Spectral formation through controlled light-source illumination represents an important technical approach for achieving the low-cost design of the instrument. During the development of the CabD light-source module, LEDs with designed center wavelengths of 650 nm, 700 nm, and 900 nm were coupled with narrow-band filters of 10 nm, 10 nm, and 16 nm, respectively, to construct Integrated Light Source 1, Integrated Light Source 2, and Integrated Light Source 3. It was therefore necessary to evaluate whether these integrated light sources could satisfy the center-wavelength and channel-bandwidth requirements for Chla and Chlb retrieval. Considering that the center wavelength and bandwidth of the integrated light sources may shift during the integration process due to the physical coupling between the LEDs and narrow-band filters (Fan et al., 2020), an ASD spectrometer was used to measure and validate their spectral characteristics. The center wavelength ( $\lambda_{c}$) and full width at half maximum (FWHM) of each channel were determined by fitting the measured spectral curves with a Gaussian function, and were subsequently compared with the spectral parameters obtained in Section 3.1 (Meier, 2005).

**Figure 11:**
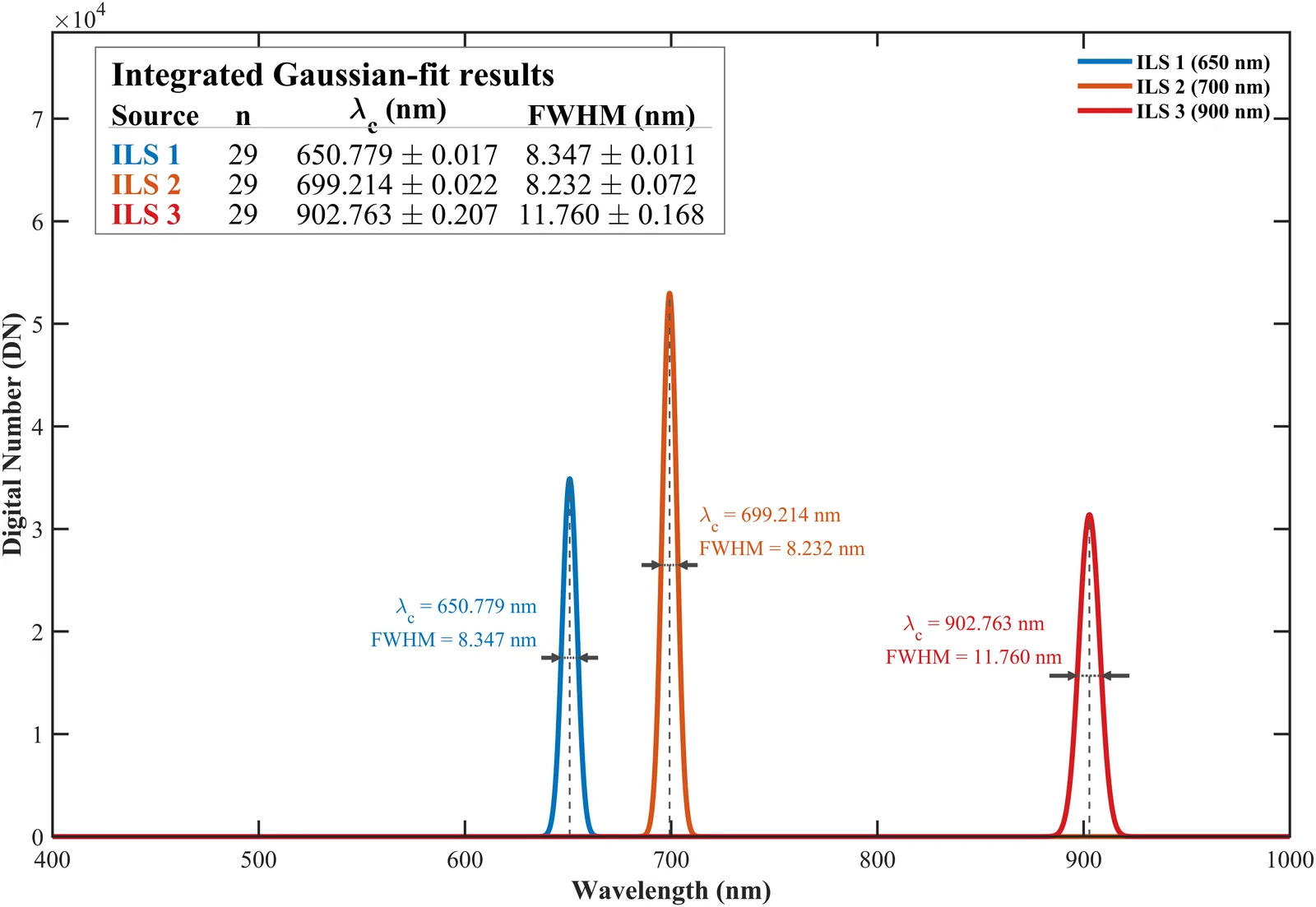
Spectral-output stability and Gaussian-fit results of the three integrated light sources during a continuous 3-h test. Each integrated light source was repeatedly measured 29 times using an ASD spectroradiometer. ILS 1, ILS 2, and ILS 3 represent the integrated light sources targeting 650, 700, and 900 nm, respectively. The vertical dashed lines indicate the fitted center wavelengths, and the horizontal bars indicate the corresponding FWHM values.

To evaluate temporal drift during continuous operation, each integrated light source was repeatedly measured 29 times using an ASD spectroradiometer over a continuous 3-h period, and the measured spectra were fitted using a Gaussian function with a constant background. The fitted center wavelengths of Integrated Light Sources 1, 2, and 3 were 650.779 ± 0.017, 699.214 ± 0.022, and 902.763 ± 0.207 nm, respectively, with corresponding FWHM values of 8.347 ± 0.011, 8.232 ± 0.072, and 11.760 ± 0.168 nm. All fitted FWHM values fell within the 6–16 nm bandwidth range selected for CabD, and the limited variations in the fitted center wavelengths and FWHM values indicated good spectral-output stability during the continuous 3-h test (**Figure 11**). With respect to center-wavelength shifts, the fitted center wavelengths of Integrated Light Sources 1, 2, and 3 deviated from their nominal values by approximately 0.8, 0.8, and 2.8 nm, respectively. These deviations were smaller than the spectral resolution of the ASD spectroradiometer, which is 3 nm, and therefore fell within the instrumental resolution limit, making their effects on the measured spectral shapes difficult to distinguish. Moreover, 699.214 and 650.779 nm remained close to the selected sensitive bands of 700 and 650 nm for Chla and Chlb retrieval, respectively, and the preceding spectral consistency analysis within the 6–16 nm bandwidth range demonstrated high spectral consistency for both bands. This further indicated that such deviations had a limited impact on retrieval performance. For Integrated Light Source 3, the fitted center wavelength of 902.763 nm was located in the near-infrared reference region, where reflectance is largely insensitive to chlorophyll absorption features. Consequently, this band exhibited stronger robustness to variations in both bandwidth and center wavelength. As shown in the previous analysis, even when the bandwidth was expanded to 30 nm, the reflectance remained highly consistent with that derived from the original 3 nm hyperspectral data (R² = 1.0000, RMSE = 0.00069). This indicated that this band primarily captured structural information rather than narrow-band absorption features. Therefore, the observed center-wavelength shift of approximately 2.8 nm, which also fell within the instrumental spectral resolution, was considered to have a limited influence on retrieval accuracy.

The measured spectral parameters of the integrated light sources, namely Integrated Light Source 1 (650.779 ± 0.017 nm, 8.347 ± 0.011 nm), Integrated Light Source 2 (699.214 ± 0.022 nm, 8.232 ± 0.072 nm), and Integrated Light Source 3 (902.763 ± 0.207 nm, 11.760 ± 0.168 nm), are in good agreement with the theoretical design targets. These results indicate that all channels meet the requirements for center wavelength and channel bandwidth in Chla and Chlb retrieval.

##### 3.2.1.2 Integrated light source reflectance normalization and stability verification

During the development of the CabD light source, the three constructed illumination channels exhibited inherent differences in sensor response due to the wavelength-dependent characteristics of LED emission intensity and optical filter transmittance. To ensure inter-channel comparability in reflectance calculation, a unified reference baseline was required for normalization.

In this study, a white reference panel was used to acquire channel-specific reference signals. Repeated white-reference measurements were averaged to obtain a fixed reference DN value for each channel. The mean DN values of Integrated Light Sources 1, 2, and 3 were 26185.1, 44464.9, and 6322.6, respectively, with corresponding RRMSE values of 0.724%, 0.954%, and 2.320% (**Table 3**). These mean DN values were stored in the embedded software as fixed white-reference parameters.

**Table 3:** Descriptive statistics of reference DN values and repeatability assessment (RRMSE) for the integrated light sources.

Light source	Max value	Min value	Mean value	RRMSE
Integrated Light Source 1	26441.1	25978.5	26185.1	0.724%
Integrated Light Source 2	44964.6	43851.9	44464.9	0.954%
Integrated Light Source 3	6564.5	6169.0	6322.6	2.320%

In subsequent processing, the leaf DN values measured by the sensor were normalized using the corresponding channel-specific reference DN values to eliminate inter-band response differences introduced by the LED–filter combinations. This normalization ensures reliable inputs for the inversion models of Chla and Chlb described in Equations 12, 13.

The integrated CabD light source meets the requirements for chlorophyll retrieval in terms of spectral parameter accuracy and light source consistency. Combined with the reference-based normalization approach, stable reflectance acquisition can be achieved without relying on complex spectroscopic systems, thereby demonstrating the feasibility of the light source-based spectral shaping strategy for low-cost Chla and Chlb sensing instruments.

#### 3.2.2 Instrument model establishment

Although the chlorophyll retrieval models derived from hyperspectral data exhibit excellent accuracy in theoretical analyses, environmental factors such as light source stability deviations, sensor response drift, reflection interference from the leaf-holding clip, and instrument dark current noise may degrade model performance in practical applications, thereby compromising their applicability and stability. Therefore, after hardware development and radiometric calibration, the embedded chlorophyll retrieval model based on the spectral overlap separation mechanism must undergo systematic calibration and reconstruction to ensure reliable detection accuracy and operational stability under real-world conditions.

During the calibration and reconstruction of the CabD chlorophyll retrieval model, the CabD dataset consisting of 162 leaf samples was used. This dataset included the reflectance spectra acquired by the developed CabD system at the characteristic bands of 650 nm, 700 nm, and 900 nm, and the reference Chla and Chlb contents determined by spectrophotometry for leaf samples collected from the same measurement positions within the instrument field of view. Repeated stratified Monte Carlo cross-validation was conducted according to a previously established method (Xu and Liang, 2001) to evaluate the stability of the CabD-specific retrieval model. In each partition, 108 samples were used to refit the device-specific retrieval coefficients using the BL-OS model structure described in Equations 12, 13, with the CabD-derived reflectance at 650 nm, 700 nm, and 900 nm as inputs, whereas the remaining 54 samples were assigned to the validation subset to evaluate the predictive performance of the recalibrated model. The corresponding calibration and validation performance distributions are presented in **Supplementary Table 3** and **Supplementary Figure 1**. Finally, the calibrated retrieval model expressions, given in Equations 19, 20, were formulated and programmed into the instrument firmware, completing the engineering transfer of the spectral overlap separation algorithm from a hyperspectral theoretical model to a low-dimensional embedded hardware platform.

(19) $$Ca=29.295\;\times \;ln(\frac{R_{900}}{R_{700}})\;-3.147\;\times \;ln(\frac{R_{900}}{R_{650}})-8.804$$

(20) $$Cb=1.513\times \;ln(\frac{R_{900}}{R_{650}})+6.211\;\times \;ln(\frac{R_{900}}{R_{700}})-2.703$$

#### 3.2.3 Instrument model accuracy verification

To verify the detection accuracy of the retrieval model recalibrated in Section 3.2.2, an accuracy assessment was conducted using the 54 CabD *in vivo* leaf samples in the validation subset of the final model. Furthermore, to evaluate the applicability of the developed CabD system under practical measurement conditions, an additional *in situ* validation dataset containing 84 intact leaf samples was collected and analyzed. Characteristic spectral signals at 650 nm, 700 nm, and 900 nm were acquired from *in vivo* leaves using the CabD instrument, and the predicted Chla and Chlb values were calculated using the recalibrated retrieval model. The corresponding reference values were determined by standard spectrophotometry using leaf samples collected from the same measurement positions. By comparing the predicted values with the reference values, RA, RMSE, and RRMSE were calculated to evaluate the predictive performance of the model and the detection accuracy of the instrument under model-validation and independent *in situ* measurement conditions.

**Figure 12:**
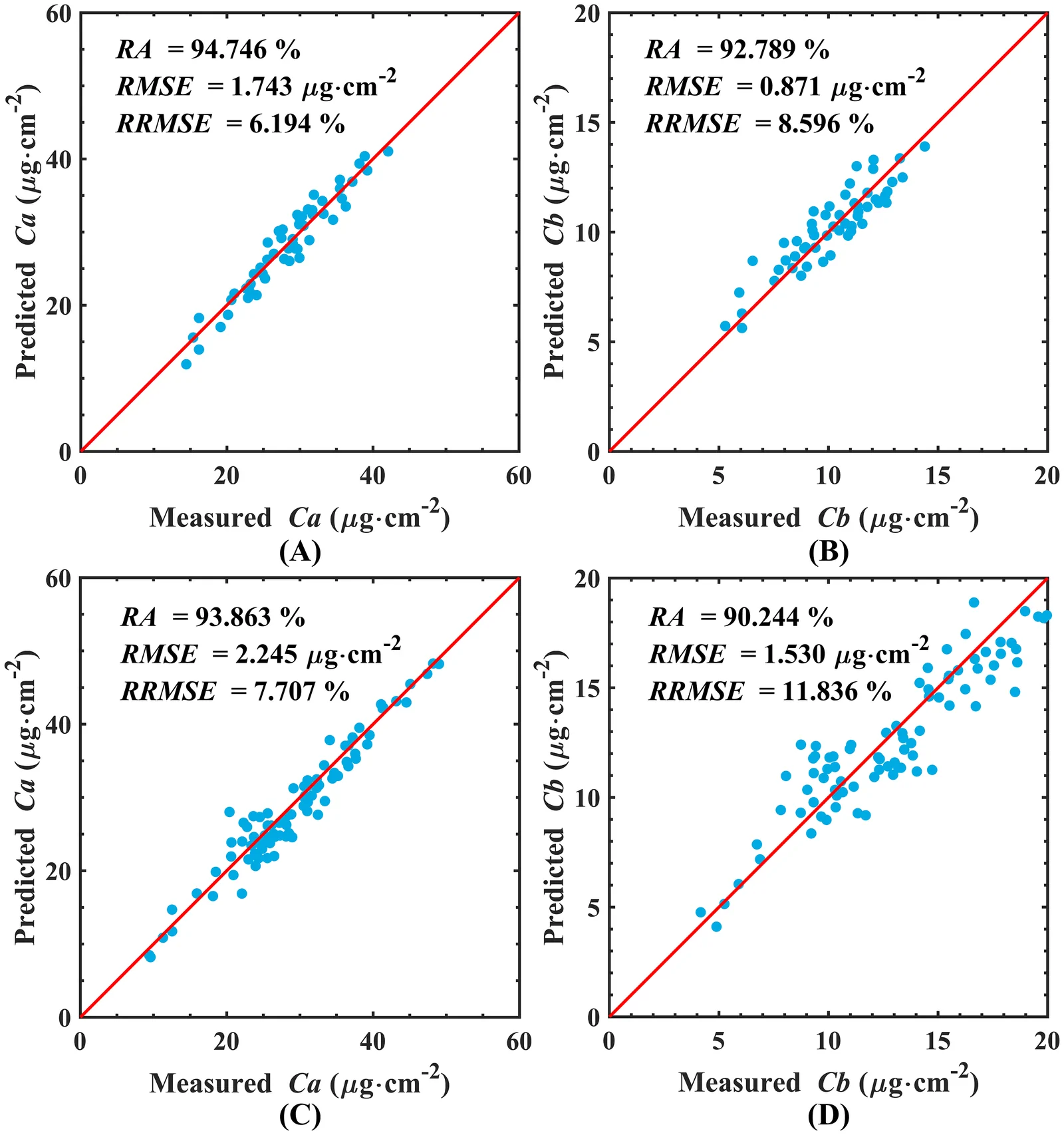
Prediction performance of CabD for Chla and Chlb under *in vivo* leaf and *in situ* validation conditions. **(A, B)** *In vivo* leaf validation; **(C, D)** *In situ* validation.

The calibration and validation performance distributions obtained from repeated cross-validation are summarized in **Supplementary Table 3**, and the validation RMSE distributions are presented in **Supplementary Figure 1**. To quantify the uncertainty of the final BL-OS and CabD-specific models, a nonparametric two-stage bootstrap analysis was conducted based on the established bootstrap framework (Efron, 1979). The validation RMSE and R² estimates and their percentile-based 95% confidence intervals are presented in **Supplementary Table 4**; **Supplementary Figure 2**. As shown in **Figure 12**, the inversion accuracy verification results demonstrate that the predicted values for Chla and Chlb show good agreement with the measured values in both *in vivo* leaf validation and *in situ* measurement datasets, with data points concentrated near the 1:1 line, indicating good consistency between predictions and measurements. For Chla (**Figure 12A**), the RMSE was 1.743 μg·cm^-2^ and the RRMSE was 6.194%, reflecting high detection consistency and a relatively low normalized prediction error. The prediction results for Chlb (**Figure 12B**) show a similar aggregation pattern, with an RMSE of 0.871 μg·cm^-2^ and an RRMSE of 8.596%, supporting the reliability of the recalibrated retrieval model. Compared with RMSE, RRMSE further normalizes the prediction error relative to the magnitude of the measured values, thereby providing a more intuitive assessment of relative prediction accuracy. The analysis indicates that the predicted values for Chla and Chlb accurately reflect the trends in the measured values, confirming the detection accuracy and reliability of the inversion model at the selected characteristic wavelengths. The results indicate that the relative accuracy (RA) for Chla reached 94.746%, and that for Chlb was 92.789%, both exceeding 90%, thus demonstrating the high-accuracy non-destructive detection capability of the instrument within the sensitive band range. To further evaluate the operational repeatability of CabD, the mean RMSE values across the 12 samples were 0.766 ± 0.486 μg·cm^-2^ for Chla and 0.288 ± 0.186 μg·cm^-2^ for Chlb, with corresponding RRMSE values of 4.240 ± 2.759% and 3.249 ± 2.114%, respectively (**Supplementary Table 5**), indicating good measurement repeatability across leaf orientations.

To further evaluate the practical applicability of CabD for non-destructive measurements, an *in situ* validation experiment was conducted using 84 intact leaf samples, and the corresponding results are presented in **Figures 12C, D**. The predicted values under *in situ* conditions also showed good agreement with the reference measurements. For Chla (**Figure 12C**), the RMSE was 2.245 μg·cm^-2^ and the RRMSE was 7.707%, while for Chlb (**Figure 12D**), the RMSE was 1.530 μg·cm^-2^ and the RRMSE was 11.836%. The relative accuracy (RA) values reached 93.863% for Chla and 90.244% for Chlb, respectively. Despite the additional variability introduced by leaf orientation, surface morphology, and the *in situ* measurement environment, the predicted values remained generally close to the 1:1 line, indicating that the CabD system retained acceptable predictive performance under practical measurement conditions.

Overall, the portable CabD showed good performance in terms of measurement accuracy, data consistency, and operational stability under both *in vivo* leaf validation and *in situ* measurement conditions, supporting its technical feasibility for practical applications. It can acquire stable spectral signals from *in vivo* leaves at 650 nm, 700 nm, and 900 nm and provide rapid predictions of Chla and Chlb contents.

## 4 Discussion

### 4.1 Construction of the BL-OS model and cost-optimized development method for low-cost spectral detectors

In this study, pseudo-absorption variables were constructed using the linearized formulation inspired by the Beer–Lambert law, revealing the differential absorption-related spectral response characteristics of Chla (sensitive band at 700 nm) and Chlb (sensitive band at 650 nm) in leaves of broadleaf species. By adopting 900 nm as the reference band for optical path length normalization *in vivo* leaf, the BL-OS framework was established for effective characterization of Chla and Chlb absorption-related responses *in vivo* leaf. The framework combines absorption-additivity information with empirical calibration for overlapping chlorophyll-related response representation.

Although it is widely recognized that multiple scattering within leaves is a major factor causing nonlinear variations in optical path length (Jacquemoud and Baret, 1990; Feret et al., 2008; Ustin et al., 2009; Xiang et al., 2025), current mainstream preprocessing strategies still rely primarily on statistical transformations such as multiplicative scatter correction (MSC) (Isaksson and Næs, 1988). However, as pointed out by Thennadil et al (Thennadil et al., 2006), such methods essentially reduce the scattering process—which contains rich physical information—to removable noise, thereby sacrificing the interpretability of the absorption spectrum. To address this limitation, this study did not adopt an elimination strategy; instead, 900 nm was introduced as a weakly absorbing reference band (Merzlyak et al., 2002) to achieve optical path length normalization for *in vivo* leaves. This reference-band normalization reduces variations in measured optical signal caused by leaf structural differences and effective optical path length under *in vivo* measurement conditions, thereby enhancing the pigment-related absorption response used in the BL-OS framework. Under this framework, the differences in spectral responses between Chla and Chlb at 700 nm and 650 nm can be effectively separated. Compared with spectral-index models that compress the mixed spectrum into a single variable, the decomposition based on pseudo-absorption coefficients substantially alleviates the spectral overlap effect between components. This is reflected by lower Chla inversion RMSE and RRMSE and comparable Chlb validation performance. Specifically, the BL-OS model yielded an RMSE of 3.001 μg·cm^-2^ and an RRMSE of 9.224% for Chla inversion, and an RMSE of 1.805 μg·cm^-2^ and an RRMSE of 12.529% for Chlb inversion. In comparison, the selected RI model for Chla and NDI model for Chlb produced higher errors, with RMSE and RRMSE values of 3.841 μg·cm^-2^ and 11.727% for Chla, and 1.950 μg·cm^-2^ and 13.536% for Chlb, respectively. This improvement observed for Chla may be attributed to the reduced overlap-related interference in the pseudo-absorption variables rather than merely to statistical fitting optimization. It is noteworthy that although Feret et al. (2008) and Zhang et al. (2022) have confirmed the physical validity of the linear superposition strategy of absorption coefficients in spectral inversion, their reliance on continuous hyperspectral data largely reflects the requirements for inversion stability and mathematical completeness, rather than necessarily arising solely from the physical constraints of leaf optical responses. Our results indicate that, with the prerequisite of 900 nm optical path normalization, the overlapping absorption-related responses of Chla and Chlb can be effectively characterized using the 650 and 700 nm sensitive bands together with the 900 nm reference band. This finding demonstrates not only the algorithmic potential of the proposed approach for the simultaneous retrieval of multiple biochemical constituents, including water content, dry matter, and anthocyanins, but also its practical value for sensor simplification. Since effective characterization can be achieved using only a few informative spectral bands, the approach may alleviate the reliance on expensive optical systems, such as hyperspectral spectrometers based on prism–grating dispersive spectral formation (Wang et al., 2024), thereby providing a physical basis for overcoming environmental constraints and developing low-cost, portable detection systems.

Based on the sensitive bands of Chla and Chlb and the reference band identified by the BL-OS inversion model, the retrieval model was further developed into the portable CabD device. Specifically, CabD employed narrow-band filters of approximately 9 nm for Integrated Light Sources 1 and 2 and approximately 12 nm for Integrated Light Source 3, in combination with controlled-illumination spectral formation, to acquire characteristic spectral signals for Chla and Chlb retrieval. The validation results showed that CabD achieved high non-destructive detection accuracy for both Chla and Chlb, with a relative accuracy (RA) of 94.746% for Chla (RMSE = 1.743 μg·cm^-2^, RRMSE = 6.194%) and 92.789% for Chlb (RMSE = 0.871 μg·cm^-2^, RRMSE = 8.596%). The *in situ* validation using 84 intact leaves yielded RMSE values of 2.245 μg·cm^-2^ for Chla and 1.530 μg·cm^-2^ for Chlb, with RA values of 93.86% and 90.24%, respectively. Despite the additional variability introduced by leaf orientation, surface morphology, and *in situ* measurement conditions, the CabD predictions remained in good agreement with the reference measurements, supporting its applicability for the *in vivo* quantification of Chla and Chlb in intact leaves.

In contrast, the conventional portable devices such as SPAD-502, CCM-200, although widely used for rapid agronomic assessment, essentially output a dimensionless ratio of transmitted light intensities related to the total chlorophyll content rather than the individual contents of Chla and Chlb *in vivo* leaf (Uddling et al., 2007; Dong et al., 2019). Constrained by the indiscriminate compression of structural interference inherent in the dual-band ratio architecture, the ratio calculation of red and near-infrared signals inevitably couples the modulation effects of leaf thickness and internal scattering (Marenco et al., 2009; Hunt and Daughtry, 2014). As revealed by Monje and Bugbee (Monje and Bugbee, 1992), light scattering and non-uniform pigment distribution fundamentally limit the *in vivo* quantitative estimation capability of such instruments. Similar optical–absolute discrepancies in leaf chlorophyll measurements under *in vivo* conditions were further discussed by Parry et al (Parry et al., 2014), who showed that the relationship between optical transmission ratios and absolute chlorophyll concentration can be nonlinear due to non-uniform chlorophyll distribution within leaves. Yang et al (Yang et al., 2009) also confirmed that variations in leaf thickness significantly interfere with the accuracy of SPAD readings. By adding a 900 nm reference integrated light source, CabD achieves optical path-length normalization at the hardware level under *in vivo* leaf conditions, thereby reducing structural and optical-path-related fluctuations in the measured optical signal and improving the stability of pigment absorption characterization. In view of the constraints of field power consumption and environmental sensitivity of spectrometers noted by Zhang et al (Zhang et al., 2025), the portable, enclosed active spectral formation architecture adopted by CabD trades off partial band continuity for the reliability required in field measurements.

This design philosophy of trading simplified optical architecture for robust physical measurements is consistent with the concept advocated by Kamarianakis and Panagiotakis (2023) in the development of low-cost chlorophyll meters.

### 4.2 Spectral bandwidth selection and re-examination of the “higher resolution is better” assumption

Although hyperspectral data are often considered to improve retrieval accuracy by enhancing spectral resolution, previous studies have shown that chlorophyll retrieval accuracy does not necessarily increase monotonically with spectral resolution (Broge and Leblanc, 2001; Dian et al., 2016; Zhai et al., 2021). Instead, retrieval performance depends jointly on spectral bandwidth, band position, signal-to-noise ratio, and the spectral characteristics of the target absorption feature. This study examined this relationship from the perspective of spectral consistency using multi-scale resampling of the original 3 nm spectra. The results indicate that within the 6–16 nm bandwidth range, the resampled spectra of each characteristic band remained highly consistent with the original spectra (with R² values close to 1 and reflectance-resampling RMSE values remaining at low levels). This suggests that within this bandwidth range, bandwidth broadening primarily results in the integration and smoothing of fine-scale spectral variations, while preserving the principal spectral patterns at the selected wavelengths. Because chlorophyll absorption in the red and red-edge regions extends over relatively broad spectral intervals, a moderate-bandwidth channel can integrate absorption-related responses across a wider wavelength range within a single spectral channel. This result is also consistent with previous work indicating that the information content of hyperspectral data is constrained by biochemical variability, canopy/leaf structural effects, and sensor characteristics, rather than by spectral resolution alone (Asner, 1998; Ustin et al., 2004). The relationship between spectral consistency and retrieval performance nevertheless remains dependent on the spectral region and measurement conditions. Moreover, the tolerance for bandwidth expansion exhibited clear band-dependent behavior: when the bandwidth was expanded to 30 nm, spectral consistency showed a clear divergence. The consistency between the 700 nm red-edge band and the original spectrum decreased markedly, while the near-infrared region remained relatively stable. Similar sensitivity differences related to spectral resolution and noise have been documented in PROSPECT-based stability evaluations, indicating that the influence of reduced spectral resolution on retrieval performance is band- and condition-dependent (Zhai et al., 2021). Therefore, the sensitivity of spectral responses to reduced resolution depends not only on the absolute bandwidth but also on the spectral structural characteristics of the band.

The differences between the RMSE values for Chla and Chlb retrieval derived from the 16 nm resampled spectral data and those from the original 3 nm hyperspectral retrieval were relatively small in this study. This observation is consistent with previous studies showing that increasing spectral resolution or narrowing spectral bands does not always produce proportional improvements in vegetation biochemical retrieval, and that retrieval accuracy is jointly constrained by bandwidth, band position, and spectral noise (Broge and Leblanc, 2001; Dian et al., 2016; Zhai et al., 2021). The results obtained from the 16 nm resampled spectra indicate that most of the spectral information required by the BL-OS model was retained after moderate bandwidth broadening. In this context, moderate bandwidths provide a balance between fine spectral detail and the integration of chlorophyll-related absorption responses over a broader wavelength interval. Based on existing studies on practical multispectral band design and vegetation-index development, spectral bands within approximately 10–20 nm have been widely used to balance information retention and measurement robustness in vegetation biochemical retrieval (Thenkabail et al., 2000; Delegido et al., 2011). The spectral bandwidths used by the CabD developed in this study were approximately 9 nm for the visible bands and 12 nm for the NIR band, which fell within the range supported by the spectral-resampling analysis. This design avoids the high cost associated with grating-based spectroscopy and enables integrated acquisition of the chlorophyll-sensitive and reference spectral responses within each channel. Meanwhile, the CabD-based retrieval yielded RMSE values of 1.743 μg·cm^-2^ and 0.871 μg·cm^-2^ for Chla and Chlb, respectively. The corresponding RRMSE values obtained from the CabD-based retrieval were lower than those obtained from the hyperspectral retrieval, decreasing from 9.224% to 6.194% for Chla and from 12.529% to 8.596% for Chlb, further indicating the reduction of relative prediction errors in the CabD-based retrieval. This result reflects a fundamental distinction between resampled and physically measured spectra: purely mathematical spectral resampling does not alter the inherent noise characteristics of the original data, whereas filter-based measurements introduce a band-integration effect that enhances the signal-to-noise ratio and yields smoother and more stable spectral responses, thereby effectively reducing the impact of noise on retrieval models. This finding further challenges the conventional assumption that “higher resolution necessarily leads to improved accuracy.” Existing research provides a reasonable explanation for this result from the perspective of absorption mechanisms. Carter (Carter, 1994) and Blackburn (Blackburn, 2007) noted that chlorophyll absorption in the red and red-edge regions exhibits a relatively broad spectral distribution rather than being confined to narrow bands. Studies based on the PROSPECT model by Jacquemoud and Baret (1990) and Zhang et al. (2022) further indicate that pigment absorption parameters represent the integrated absorption effect over a spectral range. In this context, observations with a moderate bandwidth can, to some extent, effectively capture a broader portion of the chlorophyll absorption-related response, thereby supporting stable characterization of target-component spectral features. Spectral resolution should therefore be considered together with band position, bandwidth, signal stability, and optical-system design, rather than as an isolated determinant of retrieval performance.

The design of the CabD not only matches the spectral-scale characteristics of chlorophyll absorption but also conforms to the band-design principles of practical remote sensing sensors. Meanwhile, the integration of a limited-band inversion model with controlled-illumination spectral formation reduces the reliance on complex prism-based dispersive systems while balancing optical-structure simplification, cost reduction, and chlorophyll-related spectral information retention.

### 4.3 Chlorophyll separation capability, challenges in multi-constituent extension, and application limitations

The BL-OS inversion model developed in this study demonstrated good performance for the separate retrieval of Chla and Chlb in broadleaf plants; however, it should be clarified that this separation should be understood as “effective separation.” The internal tissue structure of broadleaf leaves is generally more suitable for the assumptions of one-dimensional leaf radiative transfer, and relatively stable mesophyll scattering paths facilitate the robust expression of pigment absorption features in spectral signals (Jacquemoud and Baret, 1990; Sims and Gamon, 2002). In contrast, the needle-like morphology of coniferous plants leads to a substantially reduced effective light-intercepting area, and needles are often measured optically as fascicles, fundamentally altering photon transport paths and scattering mechanisms. In their study on the optical properties of Norway spruce needles, Malenovský et al. (2006) noted that the absorption coefficients in the PROSPECT model must be recalibrated to match the optical response of needles; similarly, Di Vittorio (2009) emphasized that the retrieval of pigments at the needle scale requires dedicated parameterization schemes. Therefore, when extending the current model to needle-leaf conditions, it is necessary to account for scale conversion by introducing an equivalent optical path parameter or a structural correction factor (e.g., effective stacking thickness or a directional accumulation function). This approach is supported by existing studies on needle radiative transfer (Smolander and Stenberg, 2003; Rautiainen et al., 2012).

At the biochemical constituent level, the ill-posedness of the inversion problem primarily arises from the spectral overlap among multiple constituents and their physiologically coordinated variations. Chlorophylls exhibit strong absorption features in the 400–700 nm visible region, but carotenoids and anthocyanins also show partially overlapping absorption bands in the same spectral range, making it difficult for a single spectral response to accurately attribute the contributions of each pigment type (Gitelson et al., 2003; Blackburn, 2007). In the near-infrared to shortwave-infrared region, water has distinct absorption peaks around 970 nm, 1450 nm, and 1940 nm, while dry matter components display broad absorption features in the 1700–2400 nm range, with water and dry matter features overlapping in some spectral intervals and exhibiting coordinated variation trends during physiological processes (Fourty and Baret, 1998). Based on an analysis of PROSPECT inversion limitations, Spafford et al. (2021) further pointed out that the additivity of absorption coefficients and the uncertainty in structural parameters substantially increase the degree of collinearity in the parameter space, leading to decreased stability of the inversion results. Therefore, within a multi-constituent extension framework, it is advisable to optimize strategies for selecting spectral sub-intervals and to adopt physical constraints or regularization methods to reduce the statistical dependence among parameters and thereby enhance the robustness of the inversion process; for example, the red-edge region can be employed for chlorophyll estimation, while shortwave-infrared wavelengths can be used to distinguish between water and dry matter.

## 5 Conclusions

In this study, a Beer–Lambert-inspired BL-OS inversion model was proposed to effectively characterize the overlapping absorption-related spectral responses of chlorophyll a and chlorophyll b *in vivo* broadleaf leaves. Specifically, pseudo-absorption variables were constructed by introducing a reference band for optical path normalization under *in vivo* leaf conditions. On this basis, spectral resampling and controlled-illumination spectral formation were further combined to establish a low-cost development method for spectral detectors targeting constituents with overlapping spectral features, and a CabD device was developed for *in vivo*, non-destructive detection of Chla and Chlb contents. The main conclusions are as follows.

1. The BL-OS model achieved effective characterization of the overlapping absorption-related spectral responses of Chla and Chlb *in vivo* broadleaf leaves. The bands at 700 nm and 650 nm were identified as the sensitive response bands for Chla and Chlb, respectively, while the 900 nm band was used as a reference band for optical path normalization under *in vivo* leaf conditions. Based on the original 3 nm hyperspectral data, the BL-OS model yielded RMSE values of 3.001 and 1.805 μg·cm^-2^ for Chla and Chlb, respectively, with corresponding RRMSE values of 9.224% and 12.529%. These results demonstrate the capability of the calibrated BL-OS model to support quantitative retrieval of chlorophyll components under *in vivo* broadleaf conditions with relatively low absolute and normalized prediction errors. Spectral resampling and consistency analyses further indicated that the 6–16 nm bandwidth range preserved the main spectral information at the selected wavelengths and provided a feasible basis for CabD filter-bandwidth selection.
2. This study established a low-cost development method for spectral detection instruments targeting constituents with overlapping spectral features. Based on the selected sensitive bands and suitable bandwidth configuration, this method uses narrow-band filters and LED-based controlled illumination to replace continuous hyperspectral acquisition with active spectral acquisition at several target bands, thereby reducing reliance on complex prism- or grating-based dispersive systems. On this basis, CabD was developed for non-destructive detection of Chla and Chlb contents *in vivo* leaves. The CabD incorporates three integrated light sources: Light Source 1 (650.779 ± 0.017 nm, 8.347 ± 0.011 nm), Light Source 2 (699.214 ± 0.022 nm, 8.232 ± 0.072 nm), and Light Source 3 (902.763 ± 0.207 nm, 11.760 ± 0.168 nm), enabling the acquisition of characteristic spectral signals for Chla and Chlb retrieval. The validation results showed that CabD achieved high detection accuracy for both Chla and Chlb under *in vivo* validation conditions, with an RMSE of 1.743 μg·cm^-2^, an RRMSE of 6.194%, and an RA of 94.746% for Chla, and an RMSE of 0.871 μg·cm^-2^, an RRMSE of 8.596%, and an RA of 92.789% for Chlb. Additional *in situ* validation using 84 intact leaves yielded an RMSE of 2.245 μg·cm^-2^, an RRMSE of 7.707%, and an RA of 93.86% for Chla, and an RMSE of 1.530 μg·cm^-2^, an RRMSE of 11.836%, and an RA of 90.24% for Chlb. These results indicate that the integration of limited target-band selection, optical path normalization under *in vivo* leaf conditions, and controlled-illumination spectral formation can maintain high non-destructive chlorophyll detection accuracy while reducing optical system complexity and instrument cost.

Further work will focus on three aspects. First, the transferability of the BL-OS model in multi-constituent retrieval and complex application scenarios should be evaluated. Since the current model is mainly based on the normalization relationship established under broadleaf conditions, equivalent optical path parameters or structural correction factors may be introduced in future studies to improve cross-leaf-type adaptation for needle-leaf species and other leaf types with distinct morphological structures and scattering properties. Second, the spectral coupling among multiple pigments and between water and dry matter should be further addressed. In the visible to shortwave-infrared regions, chlorophylls, carotenoids, and anthocyanins exhibit overlapping absorption features, while water and dry matter also show certain covariation patterns. Future studies may combine spectral sub-interval optimization, physical constraints, and regularized inversion strategies to reduce collinearity and ill-posedness in the parameter space. Third, although the continuous 3-h test demonstrated limited temporal variation in the center wavelengths and spectral bandwidths of the integrated light sources, the current validation datasets remain limited in scale, and field adaptability of the CabD should be further validated using larger independent datasets covering longer operating periods, more species, key growth stages, and uncontrolled environments to assess its long-term operational stability, component aging, and hardware robustness, as well as its practical application potential.

## Data Availability

The raw data supporting the conclusions of this article will be made available by the authors, without undue reservation.
