# Supplementary material for: An algorithm for effectively separating overlapping spectra of chlorophyll a and b in in vivo broadleaf leaves and the development of a low-cost non-destructive detector for their contents
Source: Front Plant Sci. 2026 Sep 3;17:1887710. doi: 10.3389/fpls.2026.1887710 (PMC13587621; doi:10.3389/fpls.2026.1887710)
Supplement: Supplementary file 1 [file DataSheet1.pdf]

Supplementary Material

1 Supplementary Figures and Tables

1.1 Supplementary Tables

Supplementary Table 1 | Description of plant leaf sample species.

Species No.	Common name	Genus and species	Family	Plant type	Sampling position	Leaf stage
1	Holly	*Ilex spp.*	*Aquifoliaceae*	Evergreen shrub	canopy	Y, M
2	Sweet Osmanthus	*Osmanthus fragrans*	*Oleaceae*	Fragrant shrub	canopy	Y, M, S
3	Citron	*Citrus medica*	*Rutaceae*	Fruit tree	canopy	M, S
4	Pittosporum	*Pittosporum spp.*	*Pittosporaceae*	Ornamental shrub	canopy	Y, M, S
5	Camphor tree	*Cinnamomum camphora*	*Lauraceae*	Evergreen tree	canopy	Y, S
6	Crepe Myrtle	*Lagerstroemia indica*	*Lythraceae*	Deciduous shrub	canopy	Y, M, S
7	Nanmu	*Phoebe hunanensis Hand*	*Lauraceae*	Evergreen tree	canopy	M, S
8	Tea tree	*Camellia sinensis*	*Theaceae*	Evergreen tree	canopy	Y, M, S
9	Camellia	*Camellia* spp.	*Theaceae*	Evergreen tree	canopy	Y, M, S
10	Magnolia	*Magnolia denudata*	*Magnoliaceae*	Ornamental tree	canopy	Y, M, S

Note: Y = Young leaf; M = Mature leaf; S = Senescent leaf. Classification based on leaf color and expansion stage.

Supplementary Table 2 | Description of the low-cost CabD system components.

No.	Module	Component	Model /Specification	Estimated Unit Price (CNY)	Key Parameters	Description
1	Sensing	Spectral Sensor	OPT3002	¥12.00–25.00	Spectral response: 300–1000 nm; optical-power measurement range: 1.2 nW·cm^{-2} to 10 mW·cm^{-2}; 23-bit effective dynamic range; I^{2}C/SMBus interface; supply voltage: 1.6–3.6 V.	Light intensity acquisition sensor.
2	Main Control Unit	Microcontroller Unit (MCU)	STM32F103C8T6	¥8.00–15.00	Arm Cortex-M3, 72 MHz; 64 KB Flash; up to 20 KB SRAM; supply: 2.0–3.6 V; I^{2}C, USART/UART, and PWM-capable timers.	Core controller for data processing and peripheral management.
3	Optical	Optical Filter	Narrow-band filters	¥300.00–600.00	Three designed channels (center wavelength/FWHM): 650/10, 700/10, and 900/16 nm. Measured integrated-channel center wavelength/FWHM (Figure	Bandpass filters for generating target spectral channels and suppressing out-of-band light.

					11, n = 29): 650.779 ± 0.017/8.347 ± 0.011, 699.214 ± 0.022/8.232 ± 0.072, and 902.763 ± 0.207/11.760 ± 0.168 nm.	
4	Light Source	LED Source	AlGaInP LED	¥30.00–90.00	Three time-division channels at nominal wavelengths of 650, 700, and 900 nm; fixed 45° illumination geometry; sequential switching controlled by the MCU; software-controlled warm-up time >11 s before measurement.	Specific wavelength excitation light source.
5	Drive Circuit	Constant-current Drive Circuit Board	PT4115-based LED driver circuit	¥30.00–50.00	Constant-current LED drive; MCU-controlled PWM intensity adjustment; synchronized time-division channel switching; stabilization delay after each light-source switching event before valid DN acquisition.	Provides constant-current driving and PWM-based intensity control for the LED light source.
/	Power Management	Battery & Charging Board	3.3V Li-Po Battery + Charging IC	¥50.00–100.00	AMS1117-3.3 regulated 3.3 V rail for the electronics; rechargeable Li-Po supply with charging/protection board.	Rechargeable power supply for portable operation.
/	Display	OLED Screen	0.96-inch OLED with I²C interface	¥10.00–30.00	0.96-inch display; I²C interface; real-time display of Chla and Chlb estimates and instrument operating status.	Human–machine interface for real-time display of Chla/Chlb values.
/	Auxiliary	Resistors, Capacitors, & Connectors	0603/0805 package passives, pin headers, etc.	¥150.00–200.00	0603/0805 passive packages plus pin headers/connectors; component used for signal conditioning, filtering, and electrical interconnection.	Foundational passive circuit elements.
/	Wireless Communication	Wi-Fi or Bluetooth Module	ESP8266 Wi-Fi module or HC-05 Bluetooth module	¥10.00–30.00	Optional wireless link; UART connection to the STM32; ESP8266 for Wi-Fi or HC-05 for Bluetooth communication with a host computer/mobile terminal.	Enables wireless data transmission and communication with external terminals.
/	Structural	Enclosure (3D Printed)	Resin or ABS Material	¥200.00–400.00	Fully enclosed, light-shielding housing; fixed 45° illumination and surface-normal detection geometry; integrated elastic leaf clip and trigger button.	Structural housing for light shielding and protection.

Note: The indices (1–5) are consistent with the labels in Figure 5(A), representing the core internal modules (photoelectric sensor, STM32 microcontroller, narrow-band filters, LED light source, and drive circuit board). Prices listed are estimated single-unit retail prices (CNY) based on the Chinese market; bulk procurement costs may vary.

Supplementary Table 3 | Performance statistics of the BL-OS and CabD-specific models across calibration–validation partitions.

Model	Subset	Pigment	RMSE(μg·cm⁻²), mean ± SD	RRMSE(%), mean ± SD
BL-OS	Calibration	Chla	3.303 ± 0.088	10.397 ± 0.312%
BL-OS	Calibration	Chlb	1.852 ± 0.049	13.498 ± 0.394%
BL-OS	Validation	Chla	3.368 ± 0.180	10.623 ± 0.640%
BL-OS	Validation	Chlb	1.881 ± 0.099	13.735 ± 0.801%
CabD	Calibration	Chla	2.072 ± 0.083	7.288 ± 0.295%
CabD	Calibration	Chlb	1.093 ± 0.039	10.632 ± 0.378%
CabD	Validation	Chla	2.177 ± 0.164	7.740 ± 0.587%
CabD	Validation	Chlb	1.077 ± 0.085	10.527 ± 0.835%

Note: Values are presented as the mean ± standard deviation across calibration–validation partitions. The performance statistics of the final selected models used in Figures 7 and 12 are reported separately in the main text.

Supplementary Table 4 | Bootstrap statistics for the validation RMSE and R^2 of the BL-OS and CabD-specific models.

Model	Pigment	Metric	Point estimate	Bootstrap mean ± SD	95% CI
BL-OS	Chla	R^2	0.901	0.894 ± 0.024	0.835–0.932
BL-OS	Chla	*RMSE* ($\mu g \cdot cm^{-2}$)	3.001	3.038 ± 0.222	2.620–3.476
BL-OS	Chlb	R^2	0.864	0.857 ± 0.029	0.793–0.904
BL-OS	Chlb	*RMSE* ($\mu g \cdot cm^{-2}$)	1.805	1.819 ± 0.131	1.563–2.078
CabD	Chla	R^2	0.923	0.915 ± 0.023	0.861–0.950
CabD	Chla	*RMSE* ($\mu g \cdot cm^{-2}$)	1.743	1.777 ± 0.151	1.487–2.069
CabD	Chlb	R^2	0.819	0.793 ± 0.053	0.669–0.877
CabD	Chlb	*RMSE* ($\mu g \cdot cm^{-2}$)	0.871	0.903 ± 0.079	0.757–1.068

Supplementary Table 5 | Sample-level descriptive statistics and relative variability of CabD-derived Chla and Chlb across different leaf orientations.

Sample	Chla Max	Chla Min	Chla Mean	Chla RRMSE (%)	Chlb Max	Chlb Min	Chlb Mean	Chlb RRMSE (%)
1	27.203	23.731	25.793	5.609	12.380	11.051	11.847	4.747
2	23.158	20.687	21.823	4.086	10.717	10.038	10.311	2.703
3	18.635	15.740	17.239	6.226	9.186	8.013	8.553	5.165
4	16.828	13.383	15.469	8.346	8.338	7.056	7.796	6.266
5	18.235	17.226	17.639	2.147	8.817	8.466	8.587	1.636
6	20.053	18.297	19.137	2.916	9.490	8.765	9.132	2.510
7	16.347	12.615	14.740	8.477	8.185	6.757	7.556	6.271
8	20.993	17.422	18.968	6.533	9.840	8.622	9.131	4.842
9	16.137	14.863	15.315	3.382	8.078	7.603	7.765	2.477
10	15.745	15.149	15.425	1.341	7.929	7.700	7.811	1.009
12	19.083	18.362	18.809	1.314	9.141	8.880	9.042	0.989
13	19.174	18.903	19.035	0.501	9.213	9.128	9.175	0.369

Note: Five measurements were obtained for each sample under different leaf orientations. Max, Min, and Mean are expressed in $\mu g \cdot cm^{-2}$. RRMSE was calculated from the deviations of the five measurements from their corresponding sample-specific mean.

1.2 Supplementary Figures

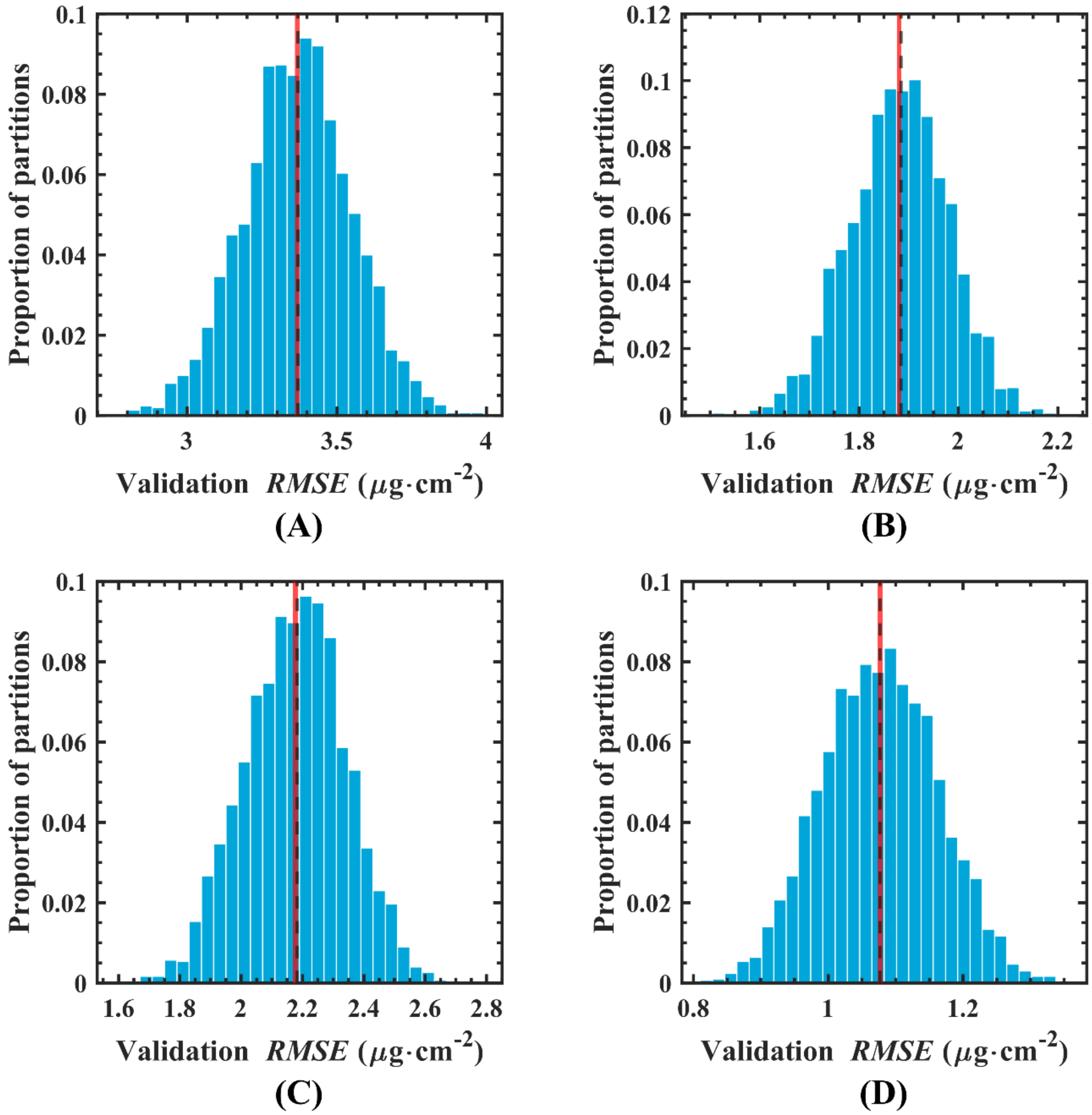

Supplementary Figure 1. Distributions of validation RMSE obtained from repeated cross-validation for (A) BL-OS Chla, (B) BL-OS Chlb, (C) CabD-specific Chla, and (D) CabD-specific Chlb models. The red solid and black dashed lines indicate the mean and median, respectively.

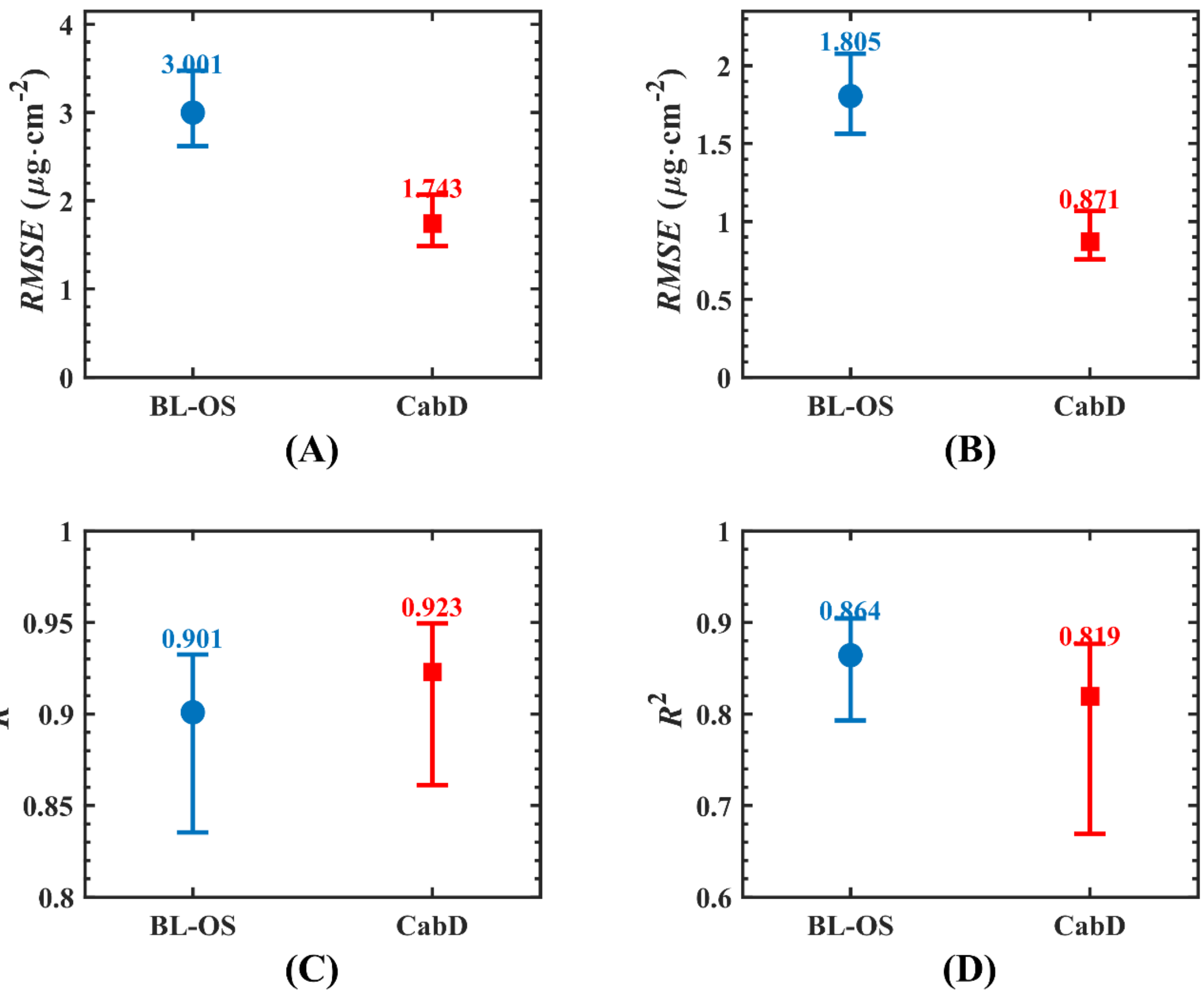

Supplementary Figure 2. Bootstrap estimates and 95% confidence intervals for (A) Chla RMSE, (B) Chlb RMSE, (C) Chla R^2, and (D) Chlb R^2. Blue circles and red squares represent the BL-OS and CabD-specific models, respectively.
